# Functional Interactions between BM88/Cend1, Ran-Binding Protein M and Dyrk1B Kinase Affect Cyclin D1 Levels and Cell Cycle Progression/Exit in Mouse Neuroblastoma Cells

**DOI:** 10.1371/journal.pone.0082172

**Published:** 2013-11-28

**Authors:** Konstantinos Tsioras, Florentia Papastefanaki, Panagiotis K. Politis, Rebecca Matsas, Maria Gaitanou

**Affiliations:** 1 Laboratory of Cellular and Molecular Neurobiology, Hellenic Pasteur Institute, Athens, Greece; 2 Center for Basic Research, Biomedical Research Foundation, Academy of Athens, Athens, Greece; Foundation for Biomedical Research Academy of Athens, Greece

## Abstract

BM88/Cend1 is a neuronal-lineage specific modulator with a pivotal role in coordination of cell cycle exit and differentiation of neuronal precursors. In the current study we identified the signal transduction scaffolding protein Ran-binding protein M (RanBPM) as a BM88/Cend1 binding partner and showed that BM88/Cend1, RanBPM and the dual specificity tyrosine-phosphorylation regulated kinase 1B (Dyrk1B) are expressed in mouse brain as well as in cultured embryonic cortical neurons while RanBPM can form complexes with either of the two other proteins. To elucidate a potential mechanism involving BM88/Cend1, RanBPM and Dyrk1B in cell cycle progression/exit, we transiently co-expressed these proteins in mouse neuroblastoma Neuro 2a cells. We found that the BM88/Cend1-dependent or Dyrk1B-dependent down-regulation of cyclin D1 is reversed following their functional interaction with RanBPM. More specifically, functional interaction of RanBPM with either BM88/Cend1 or Dyrk1B stabilizes cyclin D1 in the nucleus and promotes 5-bromo-2'-deoxyuridine (BrdU) incorporation as a measure of enhanced cell proliferation. However, the RanBPM-dependent Dyrk1B cytosolic retention and degradation is reverted in the presence of Cend1 resulting in cyclin D1 destabilization. Co-expression of RanBPM with either BM88/Cend1 or Dyrk1B also had a negative effect on Neuro 2a cell differentiation. Our results suggest that functional interactions between BM88/Cend1, RanBPM and Dyrk1B affect the balance between cellular proliferation and differentiation in Neuro 2a cells and indicate that a potentially similar mechanism may influence cell cycle progression/exit and differentiation of neuronal precursors.

## Introduction

During development of the central nervous system (CNS), coordinated regulation of cell cycle progression/exit and differentiation of neuronal precursors is essential for generation of appropriate numbers of neurons at the right time and place. Premature disruption or delayed initiation of these developmental processes alters the number of neuronal types or subtypes produced, affects neuronal connectivity and can lead to neurological dysfunction. Over the recent years, a number of studies have shown that key regulators of cell cycle progression influence neural cell fate and differentiation and conversely, cell fate determinants and differentiation-inducing proteins regulate the cell cycle [for reviews see 1-4]. 

BM88/Cend1 (hereafter Cend1) is a neuronal-lineage specific modulator implicated in coordination of cell **c**ycle **e**xit and **n**euronal **d**ifferentiation of neural stem/precursor cells [5-7]. Cend1 displays a dynamic expression pattern during central nervous system development: it is expressed at low levels in neurogenic progenitors residing at germinal layers and is upregulated as neuronal precursors exit the cell cycle and differentiate, while it persists at high levels in differentiated neurons [7,8]. Accordingly, Cend1 ceases to be expressed in neural stem/progenitor cells when they switch from a neurogenic to a gliogenic fate [8,9]. Gain-of-function approaches in neural stem/precursor cells generated from the embryonic brain and spinal cord or the postnatal subventricular zone, demonstrated that Cend1 negatively regulates proliferation, while promoting a neuronal fate [5,7]. Conversely, Cend1 silencing using RNA interference or Cend1 ablation in Cend1-null mice resulted in the opposite phenotype [7,10]. These findings suggest that Cend1 levels are important for controlling proliferation versus differentiation of neuronal precursors. The negative influence of Cend1 on cell proliferation is mediated through the cyclin D1/pRb signaling pathway that controls the balance between cell cycle progression and exit while its neuronal differentiation-promoting activity involves downregulation of Notch signaling and activation of proneural gene networks [5,7,10-12]. However, the protein partners interacting directly with Cend1 were not known. Recently, Weng et al. (2013) identified cytoplasmic Ahi1 (Abelson helper integration site-1 gene product) as a Cend1 binding protein and, in agreement with the previously published Cend1 function, showed that the interaction between Ahi1 and Cend1 influences neuronal differentiation [13]. Interestingly, Ahi1 has been associated with neurodevelopmental disorders, such as Joubert syndrome, a rare autosomal recessive disorder characterized by abnormal cerebellar development [14-17] as well as with schizophrenia and autism [18-23]. 

Cend1 cloned from porcine, mouse, human and chick brain is an integral membrane protein composed of two 22-23 kD polypeptide chains linked together by disulphide bridges [7,24,25]. Cend1 is C-tail anchored to the outer membrane of intracellular organelles, such as mitochondria, the endoplasmic reticulum and other electrolucent vesicles, with the bulk of the protein facing the cytoplasm [24-26]. Here we have identified Ran-binding protein M (RanBPM) as another direct interacting partner for Cend1 using a yeast-two hybrid system. RanBPM is a multi-domain intracellular protein that shuttles between the cytoplasm and the nucleus and has been shown to act as a scaffold for signal transduction for several receptors, nuclear proteins, transcription factors and cytoplasmic kinases in the immune and nervous systems [27]. Interestingly, RanBPM has been implicated in cell cycle progression of neuronal precursors [28] via an as yet unknown mechanism while it has been identified as a binding partner for the growth arrest protein Dyrk1B in lung epithelial cells [29]. Dyrk1B belongs to the nuclear family of **d**ual specificity t**y**rosine-phosphorylation **r**egulated **k**inases which include several vertebrate, invertebrate and lower eukaryotic orthologs characterized by highly conserved Dyrk homology and kinase domains [30,31]. Mammalian Dyrk1A and Dyrk1B and the *Drosophila* Minibrain kinases have been shown to affect proliferation and/or differentiation in a variety of cell types [30-33]. In particular, Dyrk1A has been reported to inhibit neuronal precursor cell proliferation when ectopically expressed, a function suggested to be mediated through cyclin D1 [34]. Moreover, Dyrk1B has been reported to control cyclin D1 levels in HeLa cells treated with differentiation-inducing factor-3 (DIF-3) by affecting DIF-3-induced cyclin D1 phosphorylation and degradation [35], in the lung epithelial cell line Mv1Lu [36] and in different cancer cell lines [37]. However, the expression and role of Dyrk1B in neuronal cells remain elusive.

Here we investigated the possible cross-talk between Cend1, RanBPM and Dyrk1B in cell cycle progression/exit of neuronal cells. First we showed that Cend1, RanBPM and Dyrk1B are expressed in mouse brain and in cultured embryonic cortical neurons while RanBPM can form separately complexes with either of the two other proteins when expressed in HEK293T cells. Further, by co-expression experiments in transiently transfected mouse neuroblastoma Neuro 2a cells, we found that the Cend1-dependent or Dyrk1B-dependent down-regulation of cyclin D1 is reversed following their interaction with RanBPM. More specifically, binding of RanBPM with either Cend1 or Dyrk1B stabilizes cyclin D1 in the nucleus and increases 5-bromo-2'-deoxyuridine (BrdU) incorporation as a measure of cellular proliferation. In the case of Dyrk1B-RanBPM interaction this occurs because RanBPM facilitates Dyrk1B proteasomal turnover. However, when all three proteins are co-expressed Dyrk1B is rescued in the nucleus to target cyclin D1. Additionally, co-expression of RanBPM with either BM88/Cend1 or Dyrk1B also had a negative effect on Neuro 2a cell differentiation in the presence of retinoic acid as compared with cells expressing each protein separately. Our results show that functional interactions between Cend1, RanBPM and Dyrk1B influence the balance between cellular proliferation and differentiation in Neuro 2a cells, suggesting that three proteins may also potentially play a similar role in cell cycle progression/exit and differentiation of neuronal precursors.

## Materials and Methods

### Antibodies

The affinity-purified rabbit polyclonal antibody against mouse Cend1 was raised by subcutaneous injection of GST-Cend1 chimeric protein (250 μg) in New Zealand white rabbits, once in complete and then twice in incomplete Freund’s adjuvant, followed by a final intravenous boost. Polyclonal antiserum was depleted of anti-GST antibodies by exhaustive immunopurification on nitrocellulose strips containing SDS-electrophoresed GST protein and was further immunopurified on Cend1-containing nitrocellulose strips, as previously described (Figure **S1**) [38]. The specificity of the purified antibody was verified by immunoblotting and immunocytochemistry by comparison to a previously described anti-Cend1 polyclonal antibody [24,26]. Mouse monoclonal antibody to cyclin D1 was from Santa Cruz Biotechnology (sc-450) and rabbit polyclonal antibodies against Dyrk1B (2703) and Dyrk1A (2771) were from Cell Signaling. Detection of FLAG-tagged RanBPM was performed in Western blots with mouse monoclonal anti-FLAG M2-Peroxidase conjugated (A8592) and in immunofluorescence experiments with mouse monoclonal anti-FLAG M2-FITC conjugated (F4049), all from Sigma-Aldrich. Goat polyclonal anti-RanBPM antibody (ab5295) was from Abcam. Other antibodies also used in this study were rabbit polyclonal antibodies against β-tubulin (sc-9104), α-actin and β-actin (sc-1615), mouse monoclonal antibody against βIII-tubulin (Tuj1; Covance, MMS-435P), mouse monoclonal antibody against green fluorescent protein (GFP) (Invitrogen), rabbit polyclonal against phospho-histone 3 (PH3) (Upstate) and goat polyclonal antibody against Glyceraldehyde 3-phosphate dehydrogenase (GAPDH) (Santa Cruz, sc-20357). For immunocytochemistry, Alexa-Fluor conjugated secondary antibodies were from Molecular Probes, with absorbance at 488 nm, 546 nm or 647 nm. Goat anti-GST polyclonal antibody was from GE Healthcare. 

### Plasmid Construction

**Figure 1:**
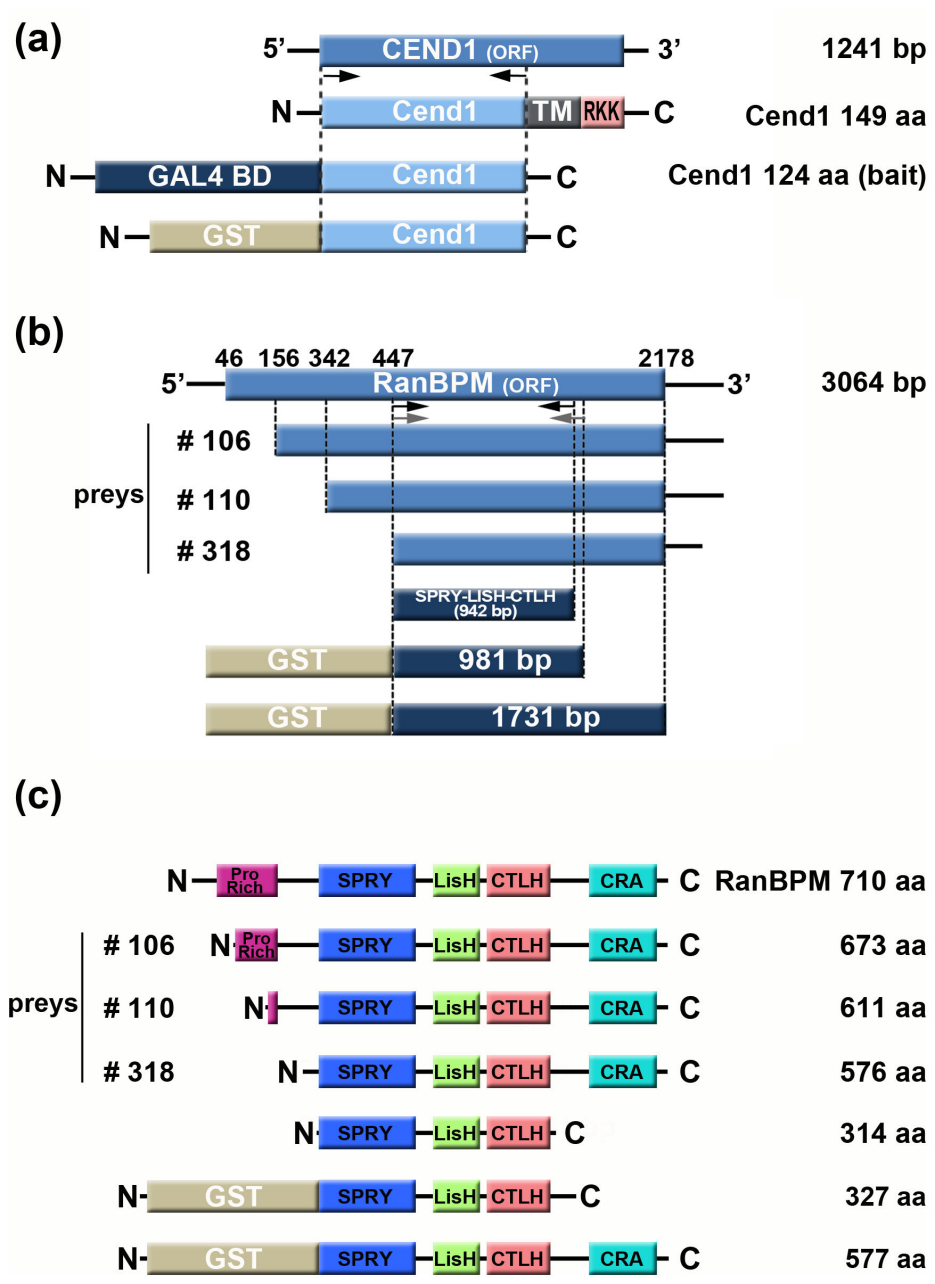
Schematic representation of Cend1 and RanBPM cDNA constructs and protein fragments. (*a*) Total mouse Cend1 cDNA (1241-bp) encodes for a 149 amino-acid protein, containing a C-terminal transmembrane region (TM) followed by RKK tail. A 375 bp fragment (372 bp plus a stop codon) corresponding to nucleotides 141 to 513 of Cend1 cDNA) and encoding the first 124 amino acids of the protein, was generated by high-fidelity PCR. This fragment was fused in frame with either the GAL4 binding domain or GST and served for yeast two hybrid screening or GST-pull down assays, respectively. (*b*, *c*) Diagrammatic illustration of the three RanBPM cDNA clones (*b*) and corresponding protein fragments (*c*) isolated from yeast two hybrid screening, all containing the multifunctional proline-rich SPRY-LISH-CTLH-CRA domain. Generation of GST-RanBPM chimeric molecules (corresponding to the 981 bp and 1731 bp cDNA fragments, as indicated) and the SPRY-LISH-CTLH domain (942 bp) was performed by high-fidelity PCR.

The mouse *Cend1* coding region, lacking the part encoding the C-terminal transmembrane domain and adjacent hydrophilic RKK tail [375bp; [24]; Figure 1a], was obtained by PCR using high fidelity Phusion DNA polymerase (Finnzymes) with primers M88FOR: 5’-CGC**GGATCC**ATGGAATCCCGAGGAAAGTC-3’ and M88REV: 5’-CCG**GAATTC**TCACAGCAAAGGGGTCAAGTT-3’. The PCR product (375 bp) was cloned into the BamHI and *EcoR I* restriction sites of the pGEX-4T.1 vector (Amersham, Pharmacia Biotech) in frame with glutathione-S-transferase (GST) and thrombin genes, and was subjected to sequence analysis for verification. This construct was used to transform the *E.coli* BL21 bacterial strain for production of GST-Cend1chimeric protein. For expression in mammalian cells, the mouse *Cend1* coding region was cloned into the pcDNA3 plasmid (Invitrogen); in some experiments a RV-pCAG-IRES-GFP construct was used in which mouse Cend1 is expressed under the control of the internal chicken β-actin promoter with cytomegalovirus enhancer (pCAG) together with GFP located after an internal ribosomal entry site (kindly generated by K. Aravantinou-Fatorou, Hellenic Pasteur Institute). Mouse GST-RanBPM constructs were generated by cloning two fragments of *RanBPM* (981 bp and 1731 bp, respectively; Figure **1b**), into *Sma I* and *Xho I* restriction sites of the pGEX-4T.1 vector using high fidelity PCR and the primers: RanBPM FORA: 5’-TCC**CCCGGG**ACGCCTCTACCCGGCTGTGGAT-3’ and RanBPM REVA: 5’-CCG**CTCGAG**TCGGCCTCCCAAACACCGCAC- 3’ for the 981 bp fragment and RanBPM FORA combined with RanBPM REVB: 5’- CCG**CTCGAG**ATGTAGGTAGTCTTCCACTGTGGCAAA-3’ for the 1731bp RanBPM fragment, respectively. A smaller cDNA fragment corresponding to the SPRY-LISH-CTLH domain of mouse RanBPM (942 bp; Figure **1b**), was also cloned by high fidelity PCR into *Xho I* and *Hind III* restriction sites of the pcDNA3A myc/His^+^ vector, using the primers RanBPM FORB: 5’-CCG**CTCGAG**ATGCGCCTCTACCCGGCTGTG-3’and RanBPM REVC: 5’-CCC**AAGCTT**TCGGCCTCCCAAACACCGCAC-3’. All clones were verified by sequencing analysis. 

### Yeast Two-hybrid Screening

The N-terminal protein fragment of mouse Cend1, which encompasses the first 124 amino acids of the protein excluding the transmembrane domain and RKK tail [[24]; Figure **1a**], was used as bait for screening the Matchmaker Gal4 Yeast Two-hybrid System 3 (Clontech). This system provides the AH109 yeast strain, which contains the reporter genes: *ADE2, HIS3, MEL1* and *lacZ*, and the Y187 yeast strain pre-transformed with the pACT2 adult mouse brain cDNA library. Mouse *Cend1* cDNA (372 bp) corresponding to the first 124 N-terminal amino acids was obtained by PCR using primers M88Y1P: 5’-AAAA**GAATTC**ATGGAATCCCGAGGAAAGTCA-3’ and M88Y2M: 5’-AAAA**CTGCAG**TCAAGGGGTCAAGTTCTCACA-3’, cloned into *EcoR I* and *Pst I* restriction sites of the pGBKT7 vector in frame with the GAL4 binding domain and subjected to sequence analysis for verification. This construct was used to transform AH109 yeast cells by the lithium acetate method according to the manufacturer’s instructions. Analysis of induction of β-galactosidase activity confirmed that the Cend1 protein fragment had no transcriptional activity by itself. The cDNA library was screened by the yeast mating method. Y189 yeast cells (MATα) containing the cDNA library, were mixed and incubated overnight with AH109 cells (MATa) containing Cend1-N124 bait, in 2X YPDA/kan^+^ medium. Diploid clones expressing interacting protein candidates were selected at high stringency conditions by plating in SD/adenine^-^/His^-^/Leu^-^/Trp^-^ medium containing 3-indolyl-D-galactopyranoside (X-α-gal) at 30°C for 6-15 days for colorimetric detection of the *MEL1* reporter gene, in the presence of 1 mM 3-amino-1, 2, 4-triasol (3-AT). Blue colonies were selected as positive candidates, grown in 3 ml of selective medium and collected by pelleting. The plasmids from candidate colonies were isolated from yeast pellets with 200 μl of 2% v/v Triton X-100, 1% v/v SDS, 100 mM NaCl, 10 mM Tris pH 8.0, 1 mM EDTA pH 8.0 and 0.3 μl glass beads followed by 200 μl phenol/chloroform/isoamyl alcohol and vortexed for 2 min at highest speed. The aqueous layer obtained after centrifugation for 5 min at 13000 rpm was used for transformation of *E.coli* ΗΒ101 bacteria. Plasmids were isolated from bacteria and subjected to sequence analysis to identify candidate interacting partners.

### GST-chimeric protein production

GST-Cend1 and GST-RanBPM fusion proteins were produced in the *E.coli* BL21 bacterial strain transformed with pGEX-4T.1-Cend1 (375 bp), pGEX-4T.1-RanBPM (981 bp) and pGEX-4T.1-RanBPM (1731 bp) (Figure **1a, b**). Protein expression in pGEX-4T.1 plasmid is under control of the *tac* promoter, which is induced by the lactose analog isopropyl β-D-thiogalactoside (IPTG). Transformed BL21 bacteria grown at 30°C with shaking until OD_600nm_ reached 0.5-2 were incubated for additional 3-6 hours after induction with 100 mM IPTG. Bacteria were then harvested and cell pellets were suspended in 20 mM Tris-HCl pH 7.5, 150 mM NaCl, 1 mM EDTA pH 8.0, 0.1% Triton X-100, containing a cocktail of protease inhibitors (1 mM PMSF, 5 μg/ml aprotinin, 5 μg/ml pepstatin A and 5 μg/ml leupeptin), followed by 5 min mild sonication on ice and addition of glycerol at a final concentration of 10%. Lysates were clarified by centrifugation at 10000 rpm for 15 min at 4°C. Clear supernatants were collected and GST-fusion proteins were purified by binding onto a 50% slurry of glutathione-Sepharose 4B (GE Healthcare) under rotation for 30 min at room temperature. Quality and yield of GST-chimeric proteins were determined by SDS-polyacrylamide gel electrophoresis and Coomassie-staining. 

### GST Pull-down Experiments

A 942 bp mouse RanBPM cDNA fragment encoding the SPRY-LISH-CTLH functional domains (Fig. **1b, c**), was cloned into the pcDNA3A myc/His^+^ plasmid (Invitrogen) and was expressed in a cell-free *in vitro* transcription-translation system (TNT Coupled Reticulocyte Lysate Systems, Promega) in the presence of 20 μCi ^35^S-methionine, using 1μg of plasmid/reaction. A fraction (40%) of the total reaction product including the ^35^S-labeled SPRY-LISH-CTLH domain of RanBPM, was incubated with 30 μg of either GST or GST-Cend1 bound onto glutathione-Sepharose 4B beads in binding buffer consisting of 20 mM HEPES pH 7.6, 150 mM NaCl, 10 mM KCl, 2.5 mM MgSO_4_, 0.5 mM 1.4-Dithio-DL-threitol (DTT), 0.1 mM ATP and 1% Nonidet P-40 (NP40). After overnight incubation on spiramix, glutathione–Sepharose 4B beads were collected by centrifugation at 500 rpm and washed six times in binding buffer before analysis by SDS-PAGE electrophoresis and autoradiography. GST-pull down assays of Cend1 from mouse brain were performed using GST-RanBPM (981 bp) and GST-RanBPM (1731 bp). Adult mouse brains were homogenized in 10 volumes of 10 mM Tris pH 7.5 containing a cocktail of protease inhibitors, and centrifuged at 3500 rpm for 15 min at 4°C. The supernatant was solubilized for 2 h at 4°C in Nonidet P-40 containing binding buffer at a dilution of 1:1 (v/v) and centrifuged at 13000 rpm for 30 min at 4°C. Clear supernatant was collected and protein concentration was measured by the DC Protein Assay kit (BioRad) according to the manufacturer’s instructions. A total amount of 15 mg mouse brain fraction was used for each GST-pull down reaction and 30 μg of either GST, GST-RanBPM (981 bp) or GST-RanBPM (1731 bp) glutathione–Sepharose 4B beads. Beads were then collected by centrifugation at 500 rpm, washed six times in binding buffer followed by SDS-PAGE electrophoresis and Western Blot analysis. For GST-pull down assays of RanBPM with GST-Cend1, Neuro 2a cells were transfected with human FLAG-tagged RanBPM full length cDNA cloned into the p3xFLAG-CMV7.1 vector (Sigma), kindly provided by Dr. Sang-Ohk Shim (Department of Neurology, School of Medicine, Yale University, USA). After 48 h of expression the cells were collected at 2000 rpm for 10 min at 4°C and the pellet was resuspended in a mild lysis buffer consisting of 10 mM HEPES pH 7.6, 150 mM NaCl, 0.1% NP40, 1 mM PMSF, 5μg/ml aprotinin, 5μg/ml pepstatin A and 5μg/ml leupeptin. For each GST-pulldown reaction 2 mg of cell lysate were mixed with 30 μg of GST or GST-Cend1 bound onto glutathione-Sepharose 4B beads and incubated O/N at 4°C. Beads were washed 6 times in mild lysis buffer and subjected to SDS-PAGE electrophoresis and Western Blot analysis.

### Cell culture and Transient Transfections

Mouse neuroblastoma Neuro 2a cells (ATCC, CCL-131) and human embryonic kidney HEK 293T cells (ATCC, CRL-11268) were maintained in Dulbecco’s modified Eagle’s medium (GIBCO-BRL) containing 10% fetal calf serum (FCS) and 1% penicillin/streptomycin (GIBCO-BRL). HEK 293T cells (3x10^6^/10-cm petri dish) or Neuro 2a cells (1.8 x 10^5^/well of a 6-well plate) were plated 1 or 2 days before transfection, respectively. Transient transfections were performed by the calcium phosphate co-precipitation method, using mouse Cend1, human FLAG-tagged RanBPM and mouse Dyrk1B full-length cDNAs. Mouse Dyrk1B cDNA (BC019545, clone identity, IMAGE: 4511845) subcloned into the mammalian expression vector pCMV-SPORT6 was obtained from the U.K. Human Genome Mapping Project Resource Centre, Hinxton, Cambridge, U.K. Cells were allowed to express proteins for 16 h or 48 h after transfection. Transfection efficiency between experiments (20-30%) was monitored in sister cultures by using an GFP expression plasmid (Clontech) for expression of enhanced GFP protein. For protein turnover assays, Neuro 2a cells were transfected with mouse Dyrk1B cDNA and allowed to express Dyrk1B protein for 16 h. Cells then were treated with cycloheximide (100 μg/ ml) for different times and subjected to Western blotting analysis. For induction of differentiation, Neuro 2a cells were exposed for 48 h to 20 μM retinoic acid (RA) in the presence of 2% FCS, as previously described [39]. The degree of cell differentiation was assessed by immunofluorescence microscopy after staining for the neuronal marker beta III-tubulin. Cells in areas of low density, bearing a neurite longer than one mean cell diameter, were scored as differentiated. One-way ANOVA was performed to examine for overall statistically significant differences among groups, followed by Student’s t-test comparison within pairs of groups.

### Primary culture of dissociated embryonic mouse cortical neurons

This study was carried out in strict compliance with the European and National Law for Laboratory Animals Use (Directive 2010/63/EU and Greek Law 161/91), with the FELASA recommendations for euthanasia and Guide for the Care and Use of Laboratory Animals of the National Institutes of Health. All animal work was conducted according to protocols approved by the Institutional Animal Care and Use Committee of the Hellenic Pasteur Institute (Animal House Establishment Code: EL 25 BIO 013). License No 2375/04-04-2012 for experimentation was issued by the Greek authorities, i.e. the Veterinary Department of the Athens Prefecture. The preparation of this manuscript was made in compliance with the ARRIVE guidelines for reporting animal research. Dissociated cortical neurons were obtained from E16.5 mouse embryos. Briefly, pregnant mice were sacrificed with isoflurane inhalation and all efforts were made to minimize suffering. The uterus and embryos were exposed, embryos were decapitated and cerebral cortices were removed, dissected, and incubated in digestion solution (0.025% trypsin in HBSS) for 15 min at 37°C. After addition of DMEM with 10% FCS, the cell suspension was triturated with a fire-polished glass pipette and centrifuged at 800 × *g* for 10 min. The cell pellet was resuspended in culture medium (Neurobasal/ B-27/ 2 mM L-glutamine/ antibiotics, all from Invitrogen), seeded onto PLL-coated glass coverslips (for immunocytochemistry) or 6-well plates (for immunoblotting) at a density of 2x10^5^ cells/cm^2^. Cells were cultured up to 8 days and neuronal purity was >90% as determined by immunofluorescence labeling for the neuronal marker betaIII-tubulin and the astroglial marker glial fibrillary acidic protein (GFAP). 

### Co-immunoprecipitation

HEK293T cells double- or triple- transfected with mouse Cend1, human FLAG-tagged RanBPM and mouse Dyrk1B expression plasmids were used for co-immunoprecipitation experiments. Cell lysates prepared as described above, were pre-cleared for 2 h at 4°C using 50 μl protein A-conjugated agarose beads (Protein A Plus, Thermo). Immunoprecipitation was performed by adding 8 μg of rabbit polyclonal anti-FLAG antibody (F7425) in 2 mg of pre-cleared cell lysates, followed by overnight rocking at 4°C. Protein A-conjugated agarose beads (50 μl) were then added and rocked for 2 h at room temperature. Following six washes in lysis buffer, proteins bound onto beads were separated by SDS-PAGE and detected by Western Blot analysis using Clean-Blot IP Detection Reagent (Thermo). 

### Immunodetection

Tissue homogenates [10% w/v in 10 mM Tris/HCl, pH 7.4, containing a cocktail of protease inhibitors [40]] were prepared from wild type C57BL/6J or Cend1 knock-out [10] mouse brains. Proteins in cell lysates or brain homogenates separated by 12% SDS-PAGE were Western blotted onto nitrocellulose (Trans-Blot, Biorad) and non-specific binding sites were blocked in 5% non-fat milk in TBS (10 mM Tris-HCl pH 8.0, 150 mM NaCl) for 30 min at 37°C. Primary and secondray antibodies were diluted in 2.5% non-fat milk in TBS and washes were performed in TBS containing 0.1% Tween 20. Peroxidase-conjugated secondary antibodies used were: goat anti-mouse and anti-rabbit IgG+IgM (H+L) (Thermo) or rabbit anti-goat whole IgG (Sigma), all diluted 1:1000 in 2.5% non-fat milk/TBS. Protein bands were detected by the enhanced chemiluminescence (ECL) Lumi-Light kit (Roche). Quantification of band intensity and normalization relative to α-actin or β-tubulin was performed by using the Image J software.

### Immunocytochemistry

Cells were washed three times with PBS and fixed with 4% paraformaldehyde for 20 min at room temperature. After blocking of non-specific binding sites for 30 min with 5% normal goat or donkey serum (Chemicon) in PBS containing, or not, 0.1% Triton X-100, cells were incubated with primary antibodies overnight at 4°C followed by 2 h incubation with appropriate secondary Alexa-Fluor antibodies with absorbance at 488 nm, 546 nm or 647 nm (Molecular Probes, Invitrogen). Nuclei were visualized by counter-staining with DAPI or TO-PRO-3 (Molecular Probes, Invitrogen). For 5-bromo-2’-deoxyuridine (BrdU) labeling, cells were given a single 1-h pulse of BrdU (100 μM) followed by fixation and immunofluorescence staining with a rat polyclonal anti-BrdU antibody (Serotec). BrdU staining was performed as previously described [8]. Specimens were viewed and digital images were taken using an Olympus Cell-R White-Field fluorescence microscope. For quantification of BrdU incorporation index at least 2000 cells were counted in total from 5-6 independent experiments. 

### Fractionation analysis

Subcellular fractions of transiently transfected or non-transfected cells were obtained after lysis in hypotonic buffer (10 mM HEPES pH 7.9, 1.5 mM MgCl_2_, 10 mM KCl, 0.5 mM DTT containing a cocktail of protease inhibitors) as previously described [40]. 

### RNA extraction and real-time RT-qPCR analysis

Total RNA was isolated from Neuro 2a cells transiently transfected with expression plasmids for mouse Cend1 or mouse Dyrk1B alone or co-transfected with Cend1 and human FLAG-tagged RanBPM; Dyrk1B and human FLAG-tagged RanBPM; Dyrk1B and GFP. RNA isolation was performed using TrizoL Reagent (Ambion). RT-PCR was carried out using 1µg RNA and reverse transcription was performed using PrimeScript RT reagent Kit (Takara). Real time quantitative PCR reactions were done with SYBR green method and run on an ABI Prism SDS 7000 machine. Data quantification was performed with the delta-delta Ct method as previously described [41,42], using GAPDH as housekeeping gene. Primer sets used were: mDyrk1B FOR: 5’-GGTCCTTTCTCTGGCTTTCC-3’ and mDyrk1B REV: 5’-TTGATGAGGTCCACCGAGA-3’, mCyclinD1 FOR: 5’-TAGGCCCTCAGCCTCACTC-3’ and mCyclinD1 REV: 5’-CCACCCCTGGGATAAAGCA-3’, mGAPDH FOR: 5’- AACTTTGGCATTGTGGAAGG-3 and mGAPDH REV: 5-GGATGCAGGGATGATGTTCT-3’.

### Statistical analysis

Statistical analysis was performed using Student’s t-test and One-way ANOVA was performed to examine for overall statistically significant differences among groups, followed by Student’s t-test comparison within pairs of groups with p-value≤0.05 considered as statistically significant (*, p-value≤0.05; **, p-value≤0.01, *** p-value≤0.001).

## Results

### Identification of RanBPM as a Cend1-Interacting Protein

**Figure 2:**
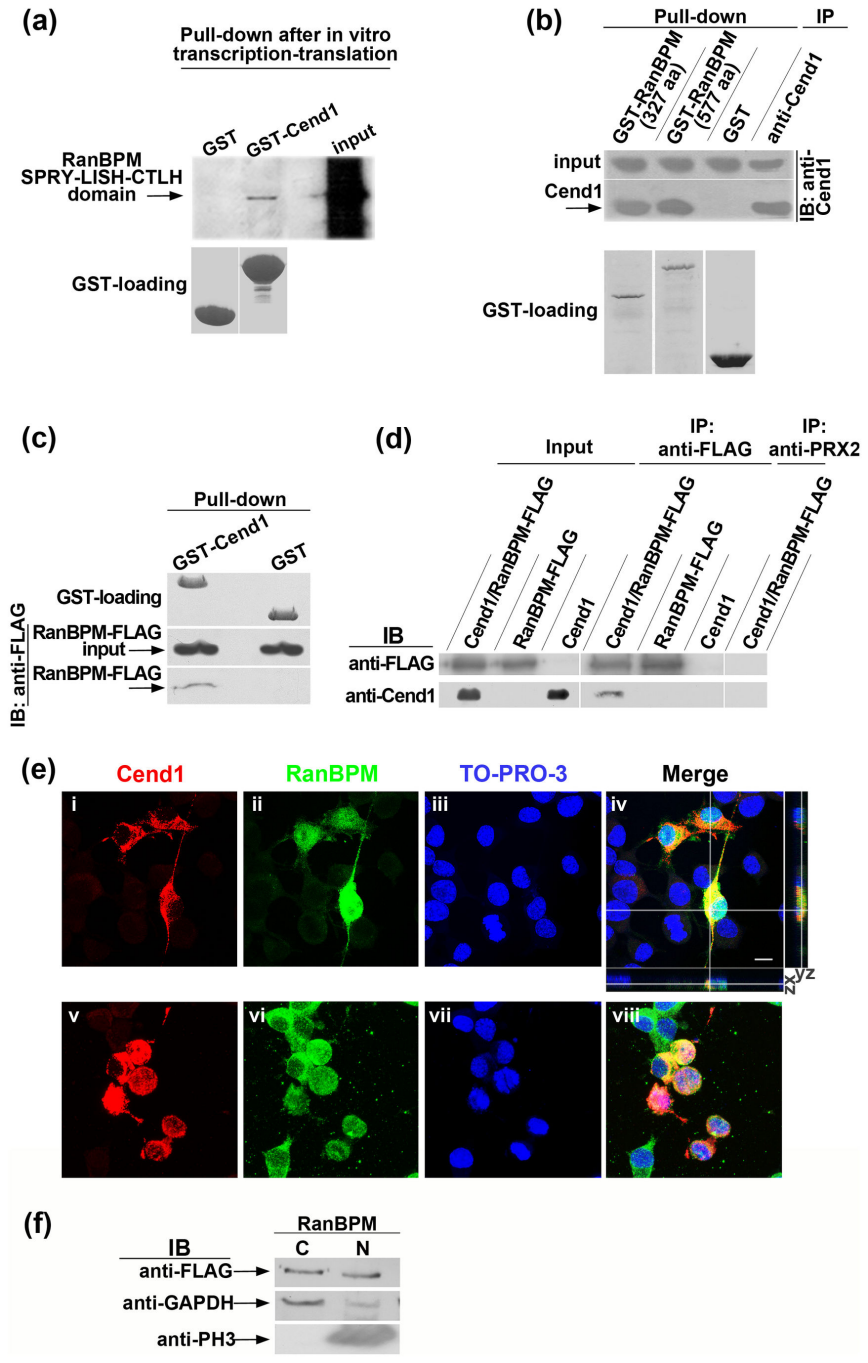
Cend1 and RanBPM interact *in* *vitro* and form complexes. (*a*) In a cell-free system, a 942 bp fragment corresponding to the SPRY-LISH-CTLH domain of RanBPM was ^35^S-labeled during coupled transcription/translation reactions and was then used in GST-pull down assays with GST-Cend1 protein or GST alone as negative control. GST-Cend1 specifically precipitates the SPRY-LISH-CTLH domain of RanBPM (upper panel). GST and GST-Cend1 protein loading is shown in the lower panel. (*b*) Reciprocal GST-pull down assays were performed in solubilized fractions of mouse brain homogenate where Cend1 is expressed in abundance (upper panel, input). GST-RanBPM (981bp) and GST-RanBPM (1731bp) fusion proteins precipitate the native Cend1 protein from mouse brain (middle panel). GST protein loading is shown in the lower panel. Cend1 immunoprecipitation with polyclonal anti-Cend1 antibody served as a positive control. (*c*) HEK 293T cells were transiently transfected with FLAG-tagged RanBPM and GST pull-down assays were performed with GST-Cend1 or GST as negative control (GST protein loading, upper panel). RanBPM (input, middle panel) was specifically precipitated by GST-Cend1 (lower panel). (*d*) Cend1 and RanBPM associate *in* *vivo* in HEK 293T cells. HEK293T cells were transiently transfected with Cend1, FLAG-RanBMP or both as indicated. Immunoprecipitation of RanBPM from HEK293T cell lysates was performed using anti-FLAG (upper panel) and detection of co-immunoprecipitated Cend1 was performed by immunoblotting using anti-Cend1 (lower panel). An irrelevant polyclonal antibody against peroxiredoxin II was used for immunoprecipitation, as negative control. (*e*) Cend1 and RanBPM co-localize in the cytoplasm of transiently co-transfected Neuro 2a cells. Double immunofluorescence labeling and confocal microscopy showed that Cend1 (red) is localized in the cytoplasm (*i*), while RanBPM (green) is partitioned in both the cytoplasm and the nucleus as detected by an anti-FLAG antibody (ii). Nuclei (blue) were visualized using TO-PRO-3 (iii) and the merged picture, including orthogonal optical sectioning, is shown in (iv). In (v-viii) shown is staining for the same antigens, but RanBPM is detected by an anti-RanBPM antibody. Scale bar: 10 μm. (*f*) Fractionation analysis of Neuro 2a cells transiently transfected with RanBPM followed by Western blot analysis, confirmed that RanBPM is distributed in both the cytoplasmic (C) and nuclear fractions (N). Fraction purity was checked using anti-GAPDH (cytoplasmic marker) and anti-PH3 (nuclear marker) antibodies, respectively.

To identify Cend1 interacting protein partners, we used a yeast-two hybrid screen. Mouse Cend1 protein, lacking the C-terminal transmembrane domain and RKK tail, was used as bait to screen an adult mouse brain cDNA library. This fragment corresponds to the part of Cend1 protein facing the cytoplasm where its interacting partners are likely to reside (Figure **1a**). Approximately 3 x 10^6^ independent clones were screened and a total of 13 positive clones were isolated. Sequence analysis and BLAST homology searches showed that three independent cDNA clones, namely #106, #110 and #318, encoded partial sequences of mouse RanBPM protein (NP_064314). All three clones were overlapping and differed in length at the 5’- terminus (Figure **1b**). The longest clone 106 lacked the cDNA region encoding the initial 37 amino acids, while the shorter clones 110 and 318 lacked cDNA regions corresponding to the initial 99 and 134 amino acids, respectively, suggesting that the initial 134 amino acids of RanBPM are not essential for interaction with Cend1 (Figure **1c**). Based on the observation that the main multi-interactive domain of RanBPM defined as SPRY-LISH-CTLH was contained in all three overlapping cDNA clones, we sought to determine whether this domain is sufficient for interaction with Cend1. To this end, we performed GST-pull down assays via an *in vitro* transcription and translation system using GST-Cend1 and ^35^S-prelabeled SPRY-LISH-CTLH domain of RanBPM (Figure **1b, c**). We found that GST-Cend1 precipitates the SPRY-LISH-CTLH domain suggesting that this is an adequate binding site for Cend1 (Figure **2a**). In order to further confirm the interaction, we proceeded to reciprocal GST-pull down assays from mouse brain homogenate using two GST-RanBPM protein fragments derived from 981 bp and 1731 bp of the mouse RanBPM cDNA (Figure **1b, c**). These two fragments correspond, respectively, to the SPRY-LISH-CTLH domain of RanBPM and the shortest cDNA clone 318 identified in the yeast-two hybrid screen (Figure **1b, c**). GST-RanBPM (981 bp) and GST-RanBPM (1731 bp) chimeric proteins, but not the negative GST control, precipitated specifically the native Cend1 protein from mouse brain homogenate (Figure **2b**). As positive control an immunoprecipitation reaction was performed, using rabbit polyclonal anti-Cend1 antibody (Figure **2b**). In addition, GST-pull down experiments were performed in cell lysates from HEK293T cells transiently transfected with FLAG-tagged full length RanBPM cDNA further confirming that GST-Cend1 precipitates RanBPM and verifying the *in vitro* interaction between the two molecules (Figure **2c**). Further, Cend1 co-immunoprecipitated with RanBPM from HEK293T cells transiently co-transfected with FLAG-tagged RanBPM and Cend1 full length cDNAs, using a rabbit polyclonal anti-FLAG antibody (Figure **2d**). To determine the specificity of the immunoprecipitation reaction an irrelevant rabbit polyclonal antibody against peroxiredoxin II (anti-PRX2) was used and no immunoprecipitated protein was detected (Figure **2d**). 

**Figure 3:**
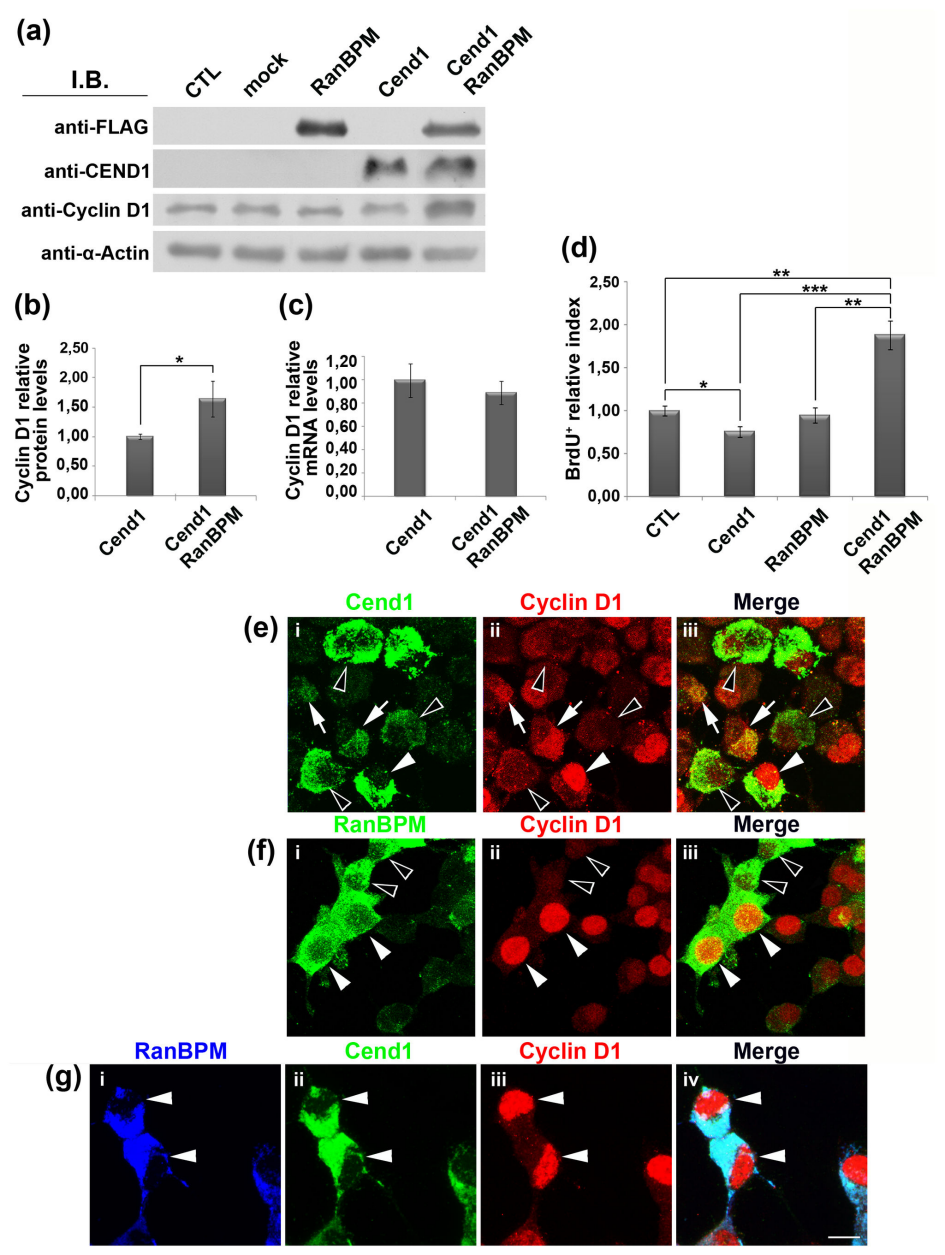
Cend1-RanBPM interaction enhances cyclin D1 levels and cell proliferation. (*a*) Western blot analysis of Neuro 2a cells transiently transfected with Cend1 or FLAG-RanBPM or both, using the indicated antibodies. An increase in cyclin D1 levels is noted upon Cend1-RanBPM co-transfection. Non-transfected Neuro 2a cells (CTL) and mock-transfected cells with empty vector were used as controls. (*b*) Quantification and normalization of cyclin D1 protein levels relatively to α-actin in Cend1 and Cend1/RanBPM transfected cells using the Image J software. (*c*) Quantification of cyclin D1 mRNA by real-time real-time RT-qPCR (d) Co-expression of RanBPM and Cend1 enhances BrdU incorporation. Estimation of the BrdU index was made by counting BrdU^+^ cells out of all cells in control cultures (CTL); BrdU^+^/Cend1^+^ cells out of all Cend1^+^ cells in Cend1-transfected cells; BrdU^+^/RanBPM^+^ out of all RanBPM^+^ cells in RanBPM-transfected cells; BrdU^+^/Cend1^+^/RanBPM^+^ cells out of all Cend1^+^/RanBPM^+^ cells in double transfected Neuro 2a cells, following a 1-hour BrdU pulse. At least 2000 cells were counted in each case and the BrdU index was calculated as the mean value from 6 independent experiments. Error bars represent SEM; ***Student’s t-test, p<0.001. (*e*- *g*) Co-expression of Cend1 and RanBPM stabilizes cyclin D1 in the nucleus. In Neuro 2a cells transiently transfected with Cend1, cyclin D1 is either extinct (open arrowheads) or exported from the nucleus to the cytoplasm (white arrows), and only occasionally maintained in the nucleus (white arrowhead) of Cend1^+^ cells (*e*). In RanBPM-transfected cultures, cyclin D1 is found in the nucleus (white arrowheads) or is extinct (open arrowheads) in RanBPM^+^ cells similar to control untransfected cells (*f*). In contrast, in all Cend1^+^/RanBPM^+^ cells cyclin D1 exhibited entirely nuclear localization (arrowheads in *g*). Scale bar: 10 μm.

Co-localization of Cend1 and RanBPM was confirmed by immunocytochemistry combined with confocal microscopy in transiently co-transfected Neuro 2a cells (Figures **2e** and **3g**). We have previously reported that Cend1 is anchored to the outer membrane of intracellular organelles, such as the mitochondria and endoplasmic reticulum, via a C-terminal transmembrane domain, with the bulk of the protein facing the cytoplasm [Figures **2e** and **3e**; [24-26]]. Compatibly with being a Cend1 interacting partner, RanBPM is found in the cytoplasm while it is also detected in the nucleus by using an anti-FLAG antibody [Figure **2e** **i-iv**; [29,43-45]]. Partitioning of RanBPM in the cytoplasm and the nucleus was also confirmed using an anti-RanBPM antibody which resulted in stronger cytoplasmic than nuclear signal (Figure **2e** **v-viii and 3f**). Distribution of RanBPM in the cytoplasmic and nuclear fractions was further demonstrated by subcellular fractionation followed by immnublotting of Neuro 2a cells transiently transfected with FLAG-tagged RanBPM cDNA (Figure **2f**). As an indication of fraction purity glyceraldehyde 3-phosphate dehydrogenase (GAPDH) and phospho-histone 3 (PH3) were used as cytoplasmic and nuclear markers, respectively (Figure **2f**)**.**

### Cend1-RanBPM interaction results in increased cyclin D1 levels and enhanced BrdU incorporation

We next explored the functional significance of RanBPM binding to Cend1 in the mouse neuroblastoma Neuro 2a cell line. We have previously shown that overexpression of Cend1 in Neuro 2a cells results in attenuation of their proliferation accompanied by a reduction in cyclin D1 levels while the opposite is observed upon Cend1 knock-down [12,46]. To gain insight into the consequences of Cend1 interaction with RanBPM in this system, Neuro 2a cells were transiently transfected with plasmids for expression of Cend1 or RanBPM or both, at a ratio of 1:1, as well as with an empty vector. Cells were collected 48 h after transfection and lysates were subjected to Western blot analysis using anti-FLAG, anti-Cend1 and anti-cyclin D1 antibodies (Figure **3a**). It was thus shown that RanBPM alone did not affect cyclin D1 levels as compared with either the parental Neuro 2a cells or cells transfected with the empty vector, while Cend1 overexpression resulted in approximately 20% cyclin D1 reduction. Remarkably however, when Cend1 and RanBPM were co-transfected, cyclin D1 levels were increased. Quantification and normalization of cyclin D1 protein levels relatively to a-actin demonstrated a 1.6-fold increase in Cend1/RanBPM co-transfected cells vs. cells transfected with Cend1 alone (Figure 3b; p=0.0226). No significant differences were found after quantification of cyclin D1 mRNA levels by real-time RT-qPCR in cells transfected with Cend1 alone or co-transfected with Cend1 and RanBPM (Figure **3c**), indicating a post-transcriptional effect of RanBPM on cyclin D1.

As cyclin D1 is an important regulator of G1 to S phase progression, our data suggest that the functional interaction of RanBPM with Cend1 enhances cell proliferation. To verify this, we estimated BrdU incorporation as a measure of cell proliferation in control cells, cells transfected with either Cend1 or RanBPM alone or cells co-transfected with both Cend1 and RanBPM (Figure **3d**). We found no significant differences between control cells and cells transfected only with RanBPM while a 25% reduction in BrdU incorporation was noted in Cend1-transfected cells as compared with controls (BrdU incorporation index: 32.15 + 1.79 % BrdU^+^ cells out of all cells in control cells vs. 30.48 + 2.79 % BrdU^+^/RanBPM^+^ cells out of all RanBPM^+^ cells, p=0.6288, n=6; vs. 24.24 + 1.94% BrdU^+^/Cend1^+^ cells out of all Cend1^+^ cells, p=0.0171, n=6). Interestingly, the BrdU incorporation index was higher by 2.5-fold in co-transfected cells as compared to cells transfected only with Cend1 (60.46 + 5.48 % BrdU^+^/Cend1^+^/RanBPM^+^ cells out of all Cend1^+^/RanBPM^+^ cells versus 24.24 + 1.94 % BrdU^+^/Cend1^+^ cells out of all Cend1^+^ cells p=0.0008, n=6, Figure **3d**). Increased BrdU incorporation in the double-transfected cells is indicative of increased proliferation although an elongation of the S-phase may be not excluded. 

Active cyclin D1 during G1 phase of the cell cycle is localized in the nucleus while it is inactivated by export to the cytoplasm where it is degraded by the proteasome [47]. Cyclin D1 was localized in the nucleus of practically all double transfected Cend1^+^/RanBPM^+^ cells further suggesting that the functional interaction between these two proteins stabilizes cyclin D1 in the nucleus (Figure **3g**). As previously published [12], in Neuro 2a cells transfected with Cend1 alone, cyclin D1 was found in the cytoplasm of approximately half of Cend1^+^ cells and only occasionally in the nucleus, while in others it was completely extinct (Figure **3e**). Finally in cells transfected with RanBPM only, cyclin D1 was either in the nucleus or extinct as in control cells (Figure **3f**). 

### Cend1, RanBPM and Dyrk1B are expressed in mouse brain and can form complexes

**Figure 4:**
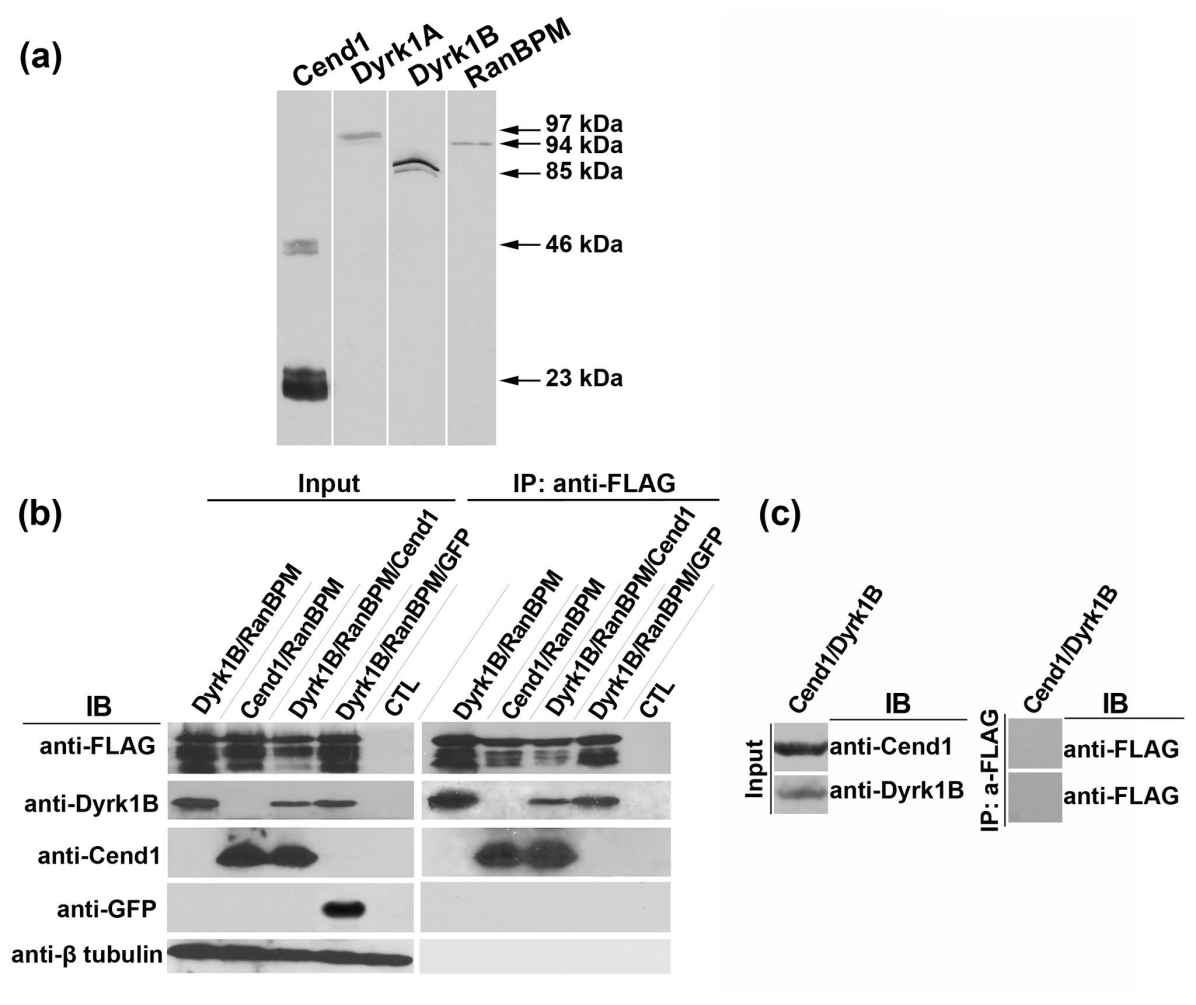
Cend1, RanBPM and Dyrk1B kinase are expressed in mouse brain and form complexes. (*a*) Mouse brain homogenate (60 μg of protein per lane) was subject to SDS-electrophoresis and immunoblotting with antibodies to Cend1 (lane 1), Dyrk1A (lane 2), Dyrk1B (lane 3) and RanBPM (lane 4). In lane 1 the 23kD band corresponding to the single polypeptide chain of Cend1 is seen as well as residual disulfide bond-linked 46kD homodimer [24,25]. (*b*) HEK293T cells were either not transfected (CTL) or transiently transfected with Cend1, RanBPM, Dyrk1B and GFP at the indicated combinations. Cells were harvested and RanBPM was immunoprecipitated from cell lysates using anti-FLAG antibody. Co-immunoprecipitated proteins were identified by Western blot using the indicated palette of antibodies. In the left panel, shown is the total cell lysate (input) with the different protein bands detected in each case. In the right panel, shown are the proteins which were co-immunoprecipitated with RanBPM in each case. (*c*) HEK293T cells were co-transfected with Cend1 and Dyrk1B and immunoprecipitated with anti-FLAG antibody. Note that anti-FLAG does not immunoprecipitate either of these proteins.

Next we explored a potential tripartite interaction between Cend1, RanBPM and the growth arrest kinase Dyrk1B in influencing cyclin D1 expression and cell cycle progression in neural cells. First we investigated Dyrk1B expression in brain by immunoblotting. We observed that Dyrk1B as well as Dyrk1A, the other family member present in brain and implicated in cell cycle progression/exit and differentiation of neuronal progenitors [32,34,48], are clearly detected in mouse brain homogenates alongside Cend1 and RanBPM (Figure **4a**). 

To examine if RanBPM co-immunoprecitates with its two identified partners Cend1 and Dyrk1B in mouse brain homogenates, we used an available goat polyclonal anti-RanBPM antibody which however could not immunoprecipitate RanBPM. Therefore, to check if the three proteins form complexes, we performed double and triple co-expression experiments with Dyrk1B, FLAG-tagged RanBPM and Cend1 in HEK293T cells, followed by immunoprecipitation using anti-FLAG antibody and Western blot analysis for detection of the precipitated proteins (Figure **4b**). Notably, we could not detect endogenous expression of any of the three proteins in HEK293T with the antibodies used in this study (data not shown). As controls, we used mock transfected cells and also a triple transfection in which Cend1 was replaced by the green fluorescent protein GFP (Figure **4b**). Finally, an additional negative control to account for RanBPM non-specific interactions was included using extracts from cells co-expressing Cend1 and Dyrk1B (Figure **4c**). Our results revealed that RanBPM co-immunoprecipitated with Dyrk1B or Cend1, respectively, from double transfected cells. Additionally, all three proteins co-immunoprecipitated from triple transfected cells (Figure **4b**). As Cend1 is localized in the cytoplasm and Dyrk1B in the nucleus the most likely interpretation of our data is that RanBPM can form independent heterodomeric RanBPM-Cend1 and RanBPM-Dyrk1B complexes. 

### RanBPM facilitates proteasomal degradation of Dyrk1B thereby stabilizing cyclin D1 levels

**Figure 5:**
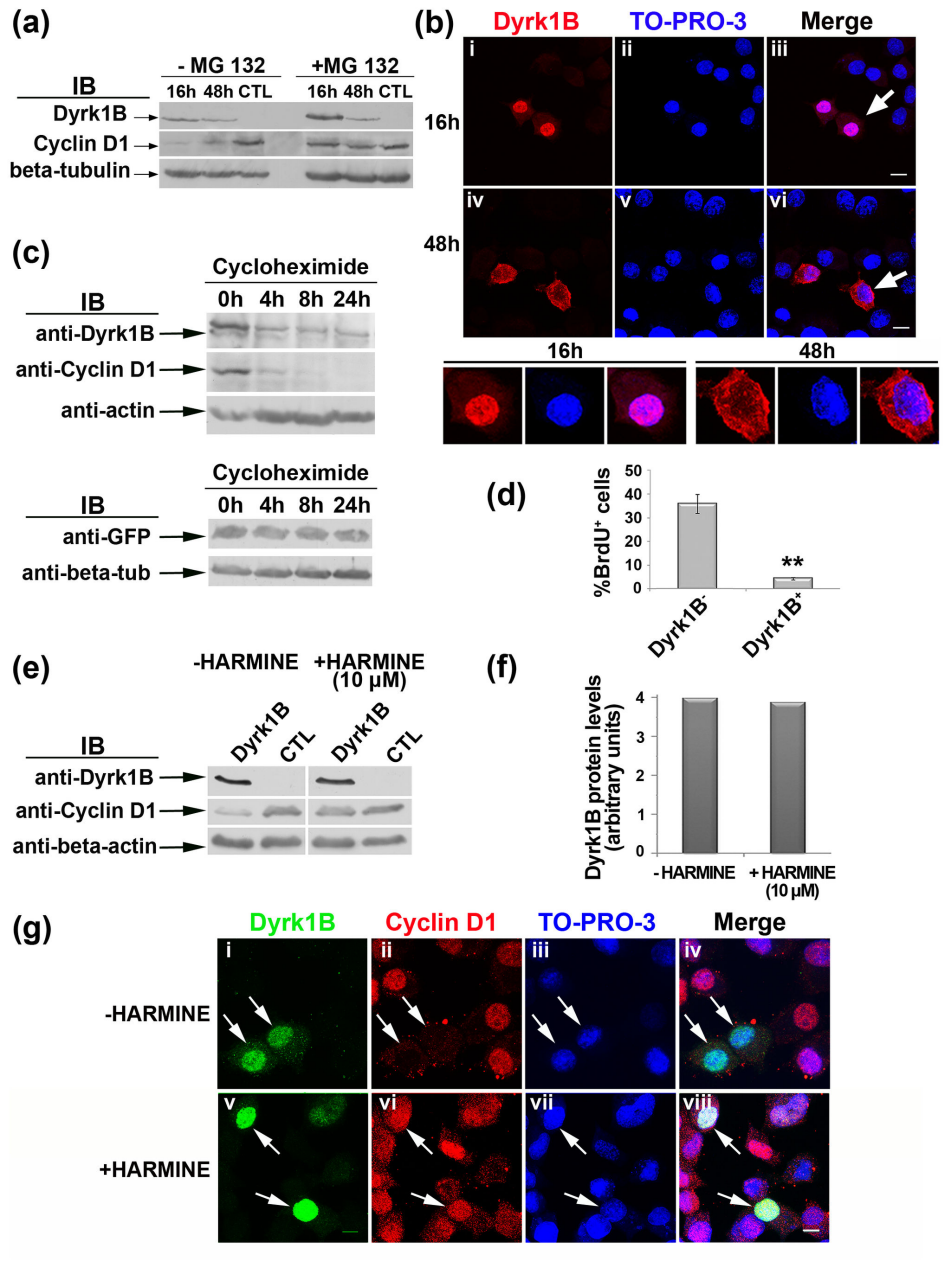
Dyrk1B protein expression and turn-over in Neuro 2a cells. (*a*) Dyrk1B expression in transiently transfected Neuro 2a cells was examined at 16 h and 48 h post-transfection in the presence or in the absence of the specific proteasome inhibitor MG132. Dyrk1B is not detectable in non-transfected Neuro 2a cells (CTL) with available antibody, while transgene Dyrk1B, obvious at 16h, is decreased overtime in a proteasome-specific manner (upper panel). In the absence of MG132, cyclin D1 levels are inversely related to Dyrk1B (middle panel) while in the presence of MG132 cyclin D1, also degraded via the proteasome, is maintained despite persisting Dyrk1B expression (middle panel); β-tubulin indicates protein loading (lower panel). (*b*) Dyrk1B is localized in the nucleus of Dyrk1B^+^ Neuro 2a cells 16 h post-transfection, while at 48 h it is mainly cytoplasmic as shown by immunocytochemistry and confocal microscopy. The cells depicted by arrows at 16h and 48h, respectively, are shown at higher magnification. Different image acquisition settings were used at 16h and 24h to compensate for signal reduction at 48h. Scale bar: 10 μm. (*c*) Dyrk1B protein turn-over in Neuro 2A cells. Cells were transfected with Dyrk1B and allowed for expression 16 h after transfection. Cells were then treated with cycloheximide for different times as indicated and subjected to Western blot analysis. (*d*) Dyrk1B reduces BrdU incorporation in transiently transfected Neuro 2a cells after 16 h of expression. **Student’s t-test, p=0.00146, n=5. (*e*) In the presence of 10 μM harmine, a specific kinase inhibitor of the Dyrk1 protein family, the Dyrk1B-dependent down-regulation of cyclin D1 is inhibited in Neuro 2a cells (middle panel) without affecting Dyrk1B protein levels (upper panel). Lanes show Neuro 2a cells transiently transfected with Dyrk1B and allowed 16 h for expression, or non-transfected cells (CTL), harvested and immunoblotted with the indicated antibodies. (*f*) Quantification of Dyrk1B protein levels normalized relative to β-actin, in the presence or absence of 10 μΜ harmine. (*g*) Cyclin D1 is wiped out from the nuclei of Dyrk1B^+^ transiently transfected Neuro 2a cells (arrows, *i*-iv) but is clearly maintained in the nucleus of Dyrk1B^+^ cells in the presence of harmine (arrows, *v*-viii). Cells were double labeled for Dyrk1B (green) and cyclin D1 (red) while nuclei were visualized using TO-PRO-3 (blue). The merged pictures are shown (iv, viii). Scale bar: 8 μm.

Next we proceeded to analyze the effect of RanBPM interaction with Dyrk1B on cell cycle progression and cyclin D1 stability, first in the absence and then in the presence of Cend1, in Neuro 2a cells. Endogenous Dyrk1B expression was not detectable by immunoblotting in non-transfected or mock transfected Neuro 2a cells cultured in standard conditions in the presence of 10% fetal bovine serum, with the antibody used in this study (Figure **5a**). In Neuro 2a cells transiently transfected with Dyrk1B cDNA, its expression was detected by immunoblotting 16 h post transfection, but declined by 48 h (Figure **5a**). This was unlike transgene Cend1 and RanBPM which remained strongly expressed at 48 h post-transfection (see Figure **3a**). Quantification by real-time RT-qPCR showed a significant decline of Dyrk1B mRNA between 16 h and 48 h, suggesting that the transgene cDNA expression became extinct over time (Figure **S3a**). 

In agreement with a function in cell cycle arrest [30], cyclin D1 was significantly reduced in Dyrk1B transfected cells at 16 h (Figure **5a**) with residual cyclin D1 being segregated in the cytosolic fraction of these cells (Figure **S2**). Notably, cyclin D1 protein increased at 48 h as Dyrk1B expression declined (Figure **5a**). Compatible with a post-transcriptional effect of Dyrk1B on cyclin D1, no significant changes were found in cyclin D1 mRNA levels between 16 h and 48 h, as quantified by real-time RT-qPCR (Figure **S3b**). 

An accumulation of cyclin D1 and to a lesser extent of Dyrk1B was observed in the presence of the 26S proteasome inhibitor MG132, included in the culture medium 6 h before cell collection, indicating that the two proteins undergo proteasomal degradation (Figure **5a**). In support, immunocytochemistry and confocal microscopy revealed that in transiently transfected Neuro 2a cells, Dyrk1B was localized in the nucleus 16 h post transfection while it was translocated to the cytoplasm at 48 h (Figure **5b**). Dyrk1B and cyclin D1 protein stability were further examined by inhibiting protein synthesis with cycloheximide. Neuro 2a cells were transfected with Dyrk1B cDNA and allowed 16h for expression after which time proteins were detected by immunoblotting before (0h) or after (4h, 8h and 24h) cycloheximide treatment (Figure **5c**). It was thus shown that Dyrk1B and more pronouncedly cyclin D1 undergo rapid protein turnover in contrast to the protein stability of GFP observed in GFP-transfected Neuro 2a cells (Figure **5c**). 

According to the above results, all further expression experiments with Dyrk1B in Neuro 2a cells were performed at 16 h post-transfection. At this time and in agreement with the Dyrk1B-induced reduction of cyclin D1 protein, the BrdU incorporation index was decreased by 8.4-fold in Neuro 2a cells transiently transfected with Dyrk1B as compared with non-transfected cells (4.23 ± 0.06% BrdU^+^/Dyrk1B^+^ cells out of all Dyrk1^+^ cells versus 35.68 ± 0.04% BrdU^+^/Dyrk1B^-^ cells out of all Dyrk^-^ cells, p=0.00146, n=5; Figure **5d**). 

It has been reported that Dyrk1B enhances the proteasomal turnover of cyclin D1 in lung epithelial cells by a mechanism involving the kinase activity of Dyrk1B which acts to phosphorylate cyclin D1 at threonine 288 [36]. To check if the same mechanism operates in Neuro 2a cells, we used harmine, a high affinity kinase inhibitor of Dyrk family members [49]. Neuro 2a cells were transfected with Dyrk1B and after removal of the transfection medium, 10 μΜ harmine was added in the culture medium and cells were allowed to express Dyrk1B for 16 h. Harmine could inhibit the Dyrk1B-dependent destabilization of cyclin D1 suggesting that the effect is kinase-dependent (Figure **5e**). Protein loading and Dyrk1B levels relative to beta-actin immunodetection were identical in the presence or absence of harmine (Figure **5e, f**). 

These data were further supported by immunocytochemistry and confocal microscopy in Neuro 2a cells transiently transfected with Dyrk1B. In the presence of harmine, cyclin D1 was stabilized in Dyrk1B^+^ nuclei of transfected cells, while in the absence of harmine, Dyrk1B^+^ nuclei were devoid of cyclin D1 (Figure **5g**). Based on the above, we conclude that Dyrk1B kinase targets cyclin D1 thus triggering its cytoplasmic translocation and proteasomal degradation. 

**Figure 6:**
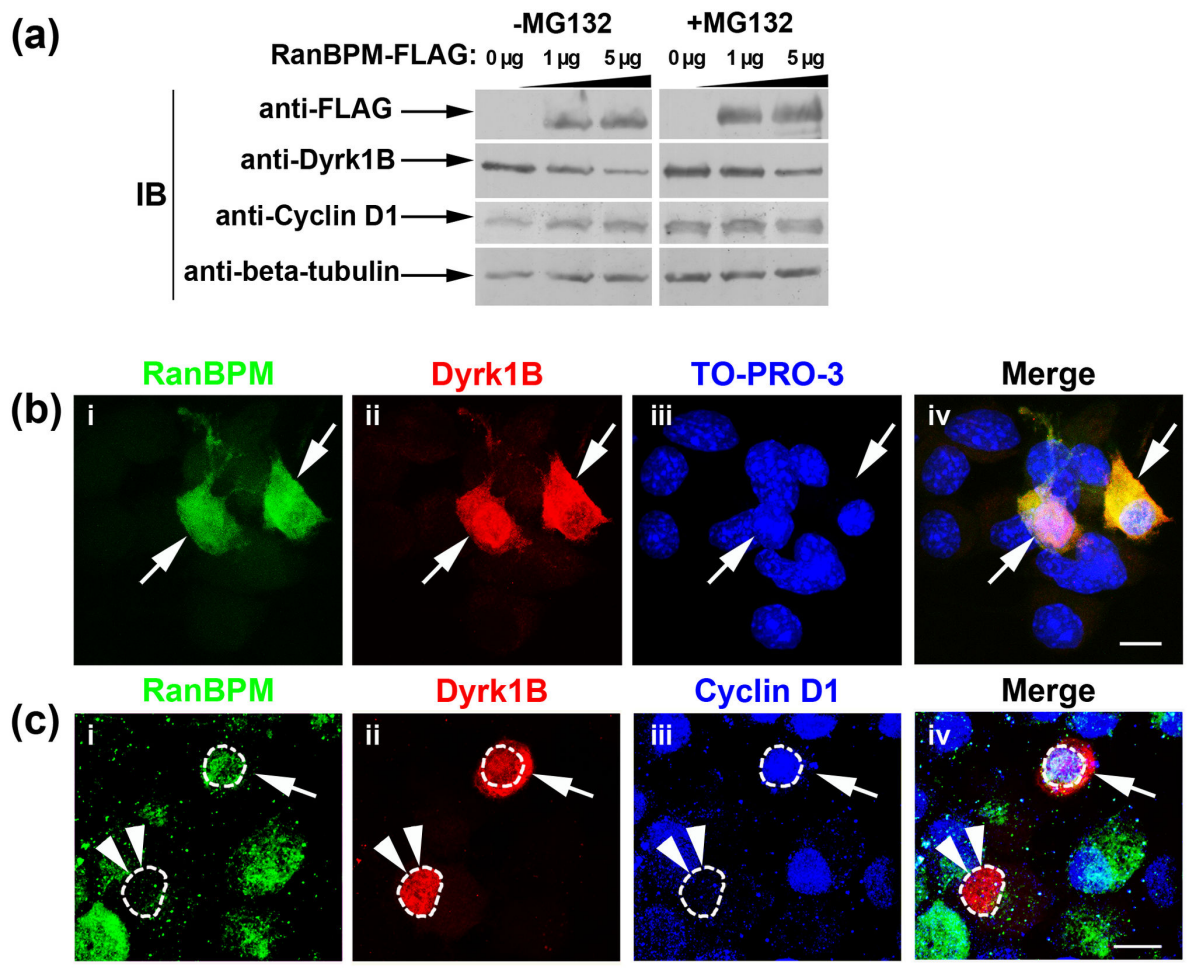
RanBPM facilitates Dyrk1B proteasomal degradation and stabilizes cyclin D1. (*a*) Neuro 2a cells were transiently co-transfected with Dyrk1B and increasing amounts of RanBPM (0, 1 and 5 μg of plasmid). Cells were maintained in culture for 16 h post transfection in the absence or in the presence of MG132 and cell lysates were subjected to Western blot analysis. In the absence of MG132, RanBPM drives Dyrk1B towards proteasomal degradation in a dose-dependent manner, while cyclin D1 levels are stabilized. When the proteasome is blocked, Dyrk1B accumulation is observed with simultaneous accumulation of cyclin D1, which is also degraded via the 26S-proteasome. (*b*, *c*) Double (*b*) and triple (*c*) immunofluorescence labeling for RanBPM (green) and Dyrk1B (red) or RanBPM (red), Dyrk1B (red) and cyclin D1 (blue). (*c*) Confocal microscopy shows that RanBPM promotes Dyrk1B cytoplasmic translocation in double positive RanBPM^+^/Dyrk1B^+^ Neuro 2a cells, 16 h post-transfection (arrows, *c*). Nuclei were visualized with TO-PRO3 (*c*). (*d*), In RanBPM^+^/Dyrk1B^+^ double positive cells, cyclin D1 is stabilized in the nucleus (arrow, d), while in cells expressing Dyrk1B, but not RanBPM (double arrowheads), cyclin D1 is not detectable in the nucleus. Scale bars: 8 μm.

Next we addressed the effect of RanBPM on Dyrk1B by co-expression of the two proteins in Neuro 2a cells for 16 h. Unexpectedly, we found that RanBPM significantly enhanced proteasomal degradation of Dyrk1B in a dose-dependent manner, a phenomenon that was inhibited by MG132 (Figure **6a**). Concomitant to the decrease in Dyrk1B, cyclin D1 levels were increased (Figure **6a**). Quantification of Dyrk1B and cyclin D1 mRNA levels by real-time RT-qPCR in Neuro2a cells co-transfected with Dyrk1B and increasing concentrations of RanBPM cDNA revealed no significant differences (Figure **S3c** **, S3d**). Similarly, in a control experiment no significant differences were noted in Dyrk1B or cyclin D1 mRNA levels in Neuro 2a cells co-transfected with Dyrk1B and increasing concentrations of GFP cDNA (Figure **S3e** **, S3f**). These results indicate that the RanBPM-induced reciprocal changes in Dyrk1B and cyclin D1 are on the protein level. 

Further, immunocytochemistry and confocal microscopy demonstrated that in the majority of double transfected RanBPM^+^/Dyrk1B^+^ cells, RanBPM facilitated translocation of Dyrk1B to the cytoplasm 16 h post transfection, at which time Dyrk1B is normally found in the nucleus (compare Figure **6b** with Figure **5b**). To address if the RanBPM-induced cytoplasmic translocation of Dyrk1B affects cyclin D1, we performed triple immunocytochemistry with antibodies against the three proteins. We found that in cells co-expressing RanBPM and Dyrk1B cyclin D1 is localized in the nucleus (Figure **6c**). Taken together, our collective immunoblot, real-time RT-qPCR and immunocytochemistry data demonstrate that RanBPM neutralizes the function of Dyrk1B on cyclin D1 by triggering Dyrk1B cytoplasmic translocation and proteasomal degradation. This results in stabilization of cyclin D1 in the nucleus, where it is presumably active to drive cell cycle progression through G1 to S-phase transition.

### Functional interactions between Cend1, RanBPM and Dyrk1B affect cyclin D1 levels in Neuro 2a cells

**Figure 7:**
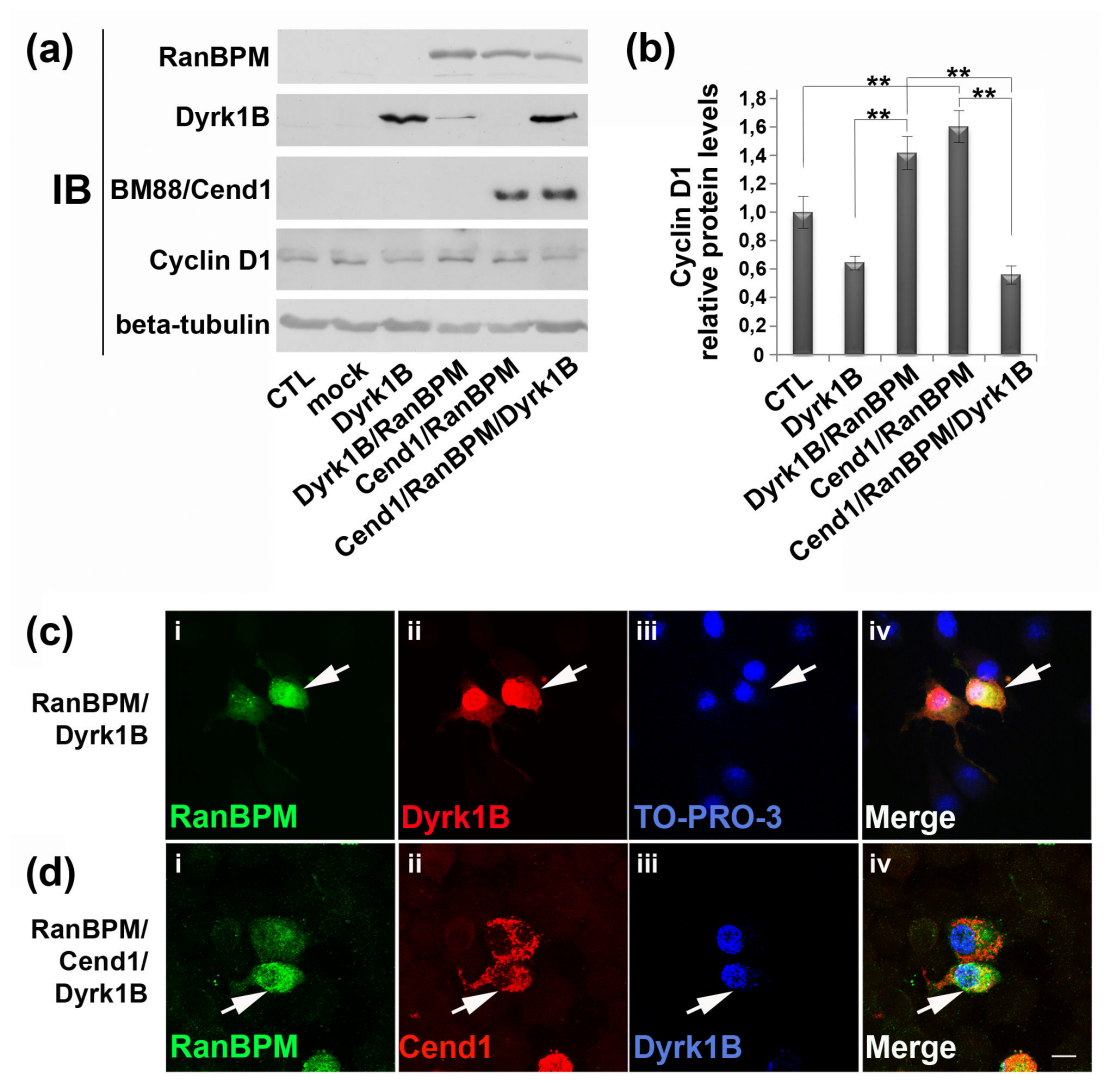
(*a*, *b*) Interactions between Cend1, RanBPM and Dyrk1B affect cyclin D1 protein levels. (*a*) Single, double or triple transient transfections with Cend1, RanBPM and Dyrk1B were performed in Neuro 2a cells, as indicated, in order to examine cyclin D1 levels. Cells were collected 16 h post-transfection and lysates were subjected to Western blot analysis (60 μg cell lysate per lane) using the indicated palette of antibodies. (*b*) Quantification of cyclin D1 protein levels normalized relative to β-tubulin was performed using the Image J software. **Student’s t-test, p<0.01, n= 10. Error bars represent SEM. Note that in double transfections with Cend1 or Dyrk1B, RanBPM caused a marked increase in cyclin D1, while in triple transfected Neuro 2a cells cyclin D1 dropped again in similar levels to those in Dyrk1B single transfected cells. (*c*, *d*) **Cend1 segregates Dyrk1B in the nucleus in the presence of RanBPM**. Double (*c*) and triple (*d*) immunofluorescence labeling and confocal analysis of Neuro 2a cells transiently transfected with RanBPM and Dyrk1B (*c*) or triple transfected with RanBPM, Dyrk1B and Cend1 (*d*), 16 h post-transfection. (*c*) RanBPM (green) facilitates Dyrk1B (red) translocation in the cytoplasm in double-transfected cells (arrows, *i*-iv). Nuclei were visualized with TO-PRO-3 (Blue, *iii*). (*d*) In triple transfected cells with RanBPM (green), Cend1 (red) and Dyrk1B (blue), Dyrk1B remains in the nucleus (arrows *iii*, iv).

Finally we assessed the impact of Cend1, RanBPM and Dyrk1B co-expression for 16 h in transiently transfected Neuro 2a cells, by monitoring cyclin D1 levels in immunoblotting experiments. Because transfected cells constituted 20-30% of the total cell population, but cyclin D1 levels were measured in the whole cell population our data represent an under-estimation of the effect of these proteins on cyclin D1 levels. As previously discussed, in double transfections with either Cend1 or Dyrk1B, RanBPM caused a marked increase in cyclin D1 (Figure **7a, b**). Quantification of cyclin D1 and normalization against beta-tubulin, revealed in Dyrk1B/RanBPM double transfected cells a statistically significant increase by 2.2-fold or 1.4-fold as compared to cells transfected only with Dyrk1B or Neuro 2a cells, respectively. On the other hand, in cells triple transfected with Cend1, Dyrk1B and RanBPM, cyclin D1 levels dropped again and were comparable to those in Dyrk1B single transfected cells (Figure **7b**). As expected Dyrk1B levels were unaffected by RanBPM in triple transfected cells (Figure **7a**). These findings suggest that when Cend1 and Dyrk1B are both present, the ability of RanBPM to stabilize cyclin D1 is eliminated. A possible explanation is that Cend1 competes effectively for RanBPM binding thus rescuing Dyrk1B in the nucleus. To explore this possibility we followed the localization of Dyrk1B by immunocytochemistry in Neuro 2a cells double-transfected with RanBPM and Dyrk1B or triple transfected with RanBPM, Dyrk1B and Cend1. As discussed above, RanBPM facilitated Dyrk1B translocation in the cytoplasm in double-transfected cells after 16 h of expression (Figures **6b** and **7c**). In contrast, in Neuro 2a cells co-expressing the three proteins, Dyrk1B was still detected in the nucleus after 16 h (Figure **7d**), but also after 48 h of expression (data not shown), indicating that in the presence of both RanBPM and Cend1 Dyrk1B is stabilized in the nucleus. These results are compatible with the hypothesis that binding of Cend1 to RanBPM rescues Dyrk1B in the nucleus. 

### Interaction of RanBPM with Dyrk1B or Cend1 affects Neuro 2a cell differentiation

**Figure 8:**
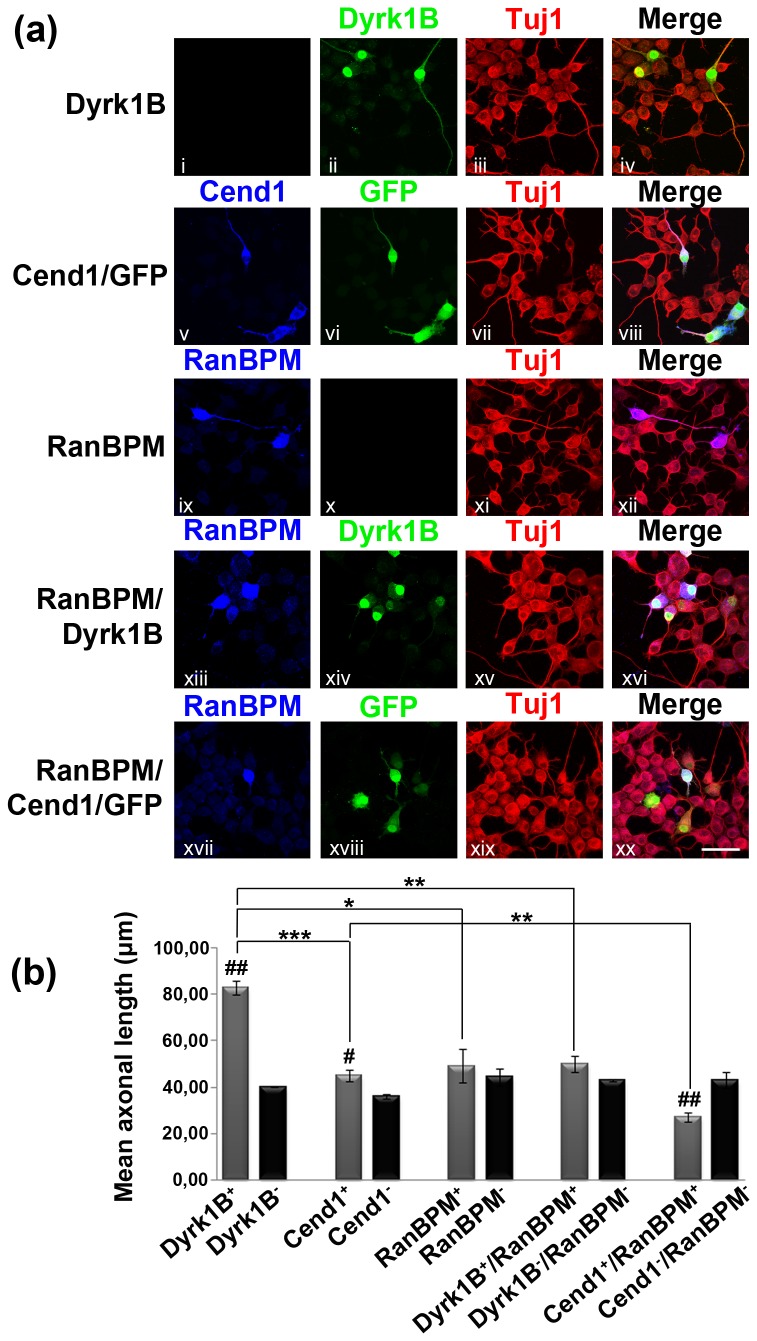
Interaction of RanBPM with Dyrk1B or Cend1 affects negatively Neuro 2a cell differentiation. (*a*) Neuro 2a cells were transiently single- and double-transfected with: Dyrk1B, pCAG-Cend1-IRES-GFP, RanBPM, RanBPM and Dyrk1B, RanBPM and pCAG-Cend1-IRES-GFP expression plasmids, as indicated. Cells were allowed 16 h for protein expression and were then induced to differentiate by treatment with 20 μM RA/ 2% FCS for 48 h after which they were fixed and immunofluorescently labeled for Dyrk1B, Cend1, RanBPM and the neuronal differentiation marker βIII-tubulin (Tuj1 antibody). Note that Cend1 is co-expressed with GFP (v, vi) which serves for visualization of Cend1-positive cells (vi, xviii). Scale bar: 40 μm. (*b*) Quantification of the mean neurite length in single and double-transfected cells (as indicated) as well as in non-transfected cells in each group. Different groups were compared by one-way ANOVA followed by Student's t-test. ***Student’s t-test: p<0.001, **: p<0.01, *: p<0.05, n= 3. Error bars represent SEM. #: represents statistically significant differences between transfected and non-transfected cells in the same group, while *: represents statistically significant differences among different groups.

As cell cycle progression/exit is tightly coupled to cell differentiation, we examined the potential effect of RanBPM binding to either Dyrk1B or Cend1 on Neuro 2a cell differentiation. Cells were transiently transfected with: a) the plasmid for expression of Dyrk1B; b) the pCAG-Cend1-IRES-GFP plasmid driving expression of both Cend1 and GFP; c) double-transfected with Dyrk1B and RanBPM and d) double-transfected with pCAG-Cend1-IRES-GFP and RanBPM. Cells were allowed 16 h for protein expression and were then induced to differentiate by treatment with 20μM RA/ 2% FCS for 48 h after which they were fixed and immunolabeled for Dyrk1B, Cend1, RanBPM and the neuronal differentiation marker βIII-tubulin (Tuj1) (Figure **8a**). In two independent experiments we observed that in single-transfectants the average differentiation index (i.e. the percentage of cells that expressed the transgene and had differentiated) was 68 % for the Dyrk1B-expressing cells and dropped to 38 % for the Dyrk1B^+^/RanBPM^+^ double positive cells (> 40% reduction). Moreover in the case of Cend1 single-transfected cells, the differentiation index was 59.5 ± 2.6 % and dropped significantly to 30.2 ± 2.2 % in the Cend1^+^/RanBPM^+^ double-positive cells (n=3, p= 0.00097) highlighting an approximately 50% reduction in differentiation. We then proceeded to estimate mean neurite length in each situation, both within the transfected as well as within the non-transfected populations (Figure **8b**). We found that the mean neurite length of Dyrk1B^+^ cells was significantly higher than that of the corresponding non-transfected cells (83.21 ± 2.99 μm vs. 40.14 ± 0.17 μm; n=3, p= 0.00481). A smaller, but statistically significant increase, was also noted in the mean neurite length of Cend1^+^ cells as compared with non-transfected cells (44.97 ± 2.59 μm vs. 36.23 ± 0.94 μm; n=3, p= 0.05) while there was no significant difference between RanBPM^+^ cells and non-transfected cells (p= 49.18 ± 7.28 μm vs. 44.31 ± 3.74 μm; n=3, p= 0.59332). When compared by single-factor ANOVA the non-transfected cells showed no significant difference between the different groups. On the other hand, in cells co-expressing Dyrk1B and RanBPM there was a dramatic reduction in the mean neurite length as compared with the Dyrk1B^+^ single transfectants, reaching almost that of non-transfected cells (83.21 ± 2.99 μm in Dyrk1B^+^ cells vs. 50.03 ± 3.28 μm in Dyrk1B^+^/RanBPM^+^ cells; n=3, p= 0.00171; compare with the value of non-transfected cells; Figure **8b**). Similarly, a significant reduction in the mean neurite length was observed in the Cend1^+^/RanBPM^+^ double transfectants as compared with the single-transfected Cend1^+^ cells (mean neurite length 44.97 ± 2.59 μm vs. 27.11 ± 2.19 μm; n=3, p= 0.00628; Figure **8b**). Our data indicate that Dyrk1B and Cend1 influence positively cell differentiation whereas the interaction of RanBPM with Dyrk1B or Cend1 has a negative effect on cell differentiation.

### Expression of Cend1, Dyrk1B and RanBPM in embryonic cortical neurons in culture

**Figure 9:**
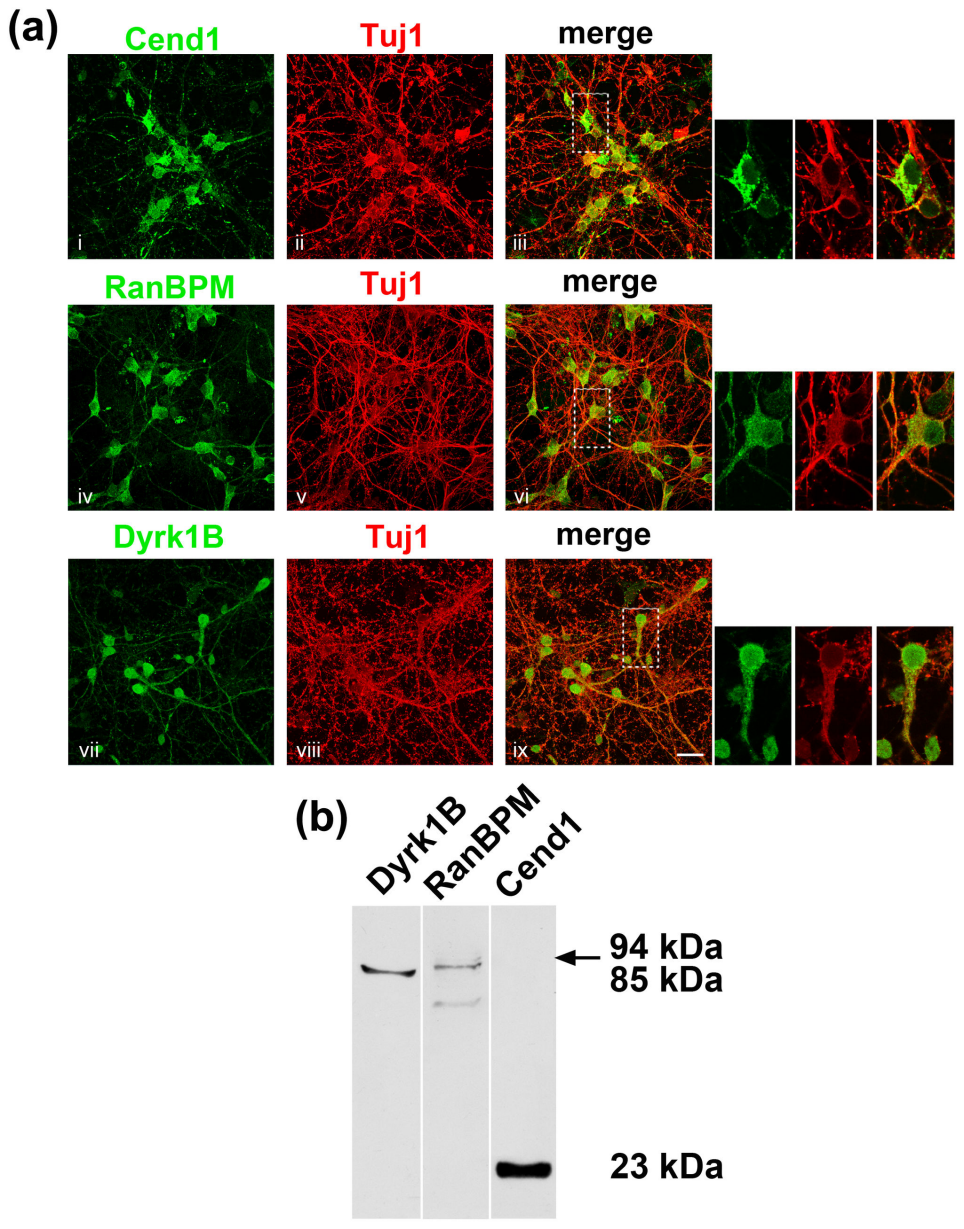
Embryonic cultured cortical neurons express Cend1, RanBPM and Dyrk1B. (*a*) Immunofluorescence labeling and confocal analysis of cortical neurons from E16.5 mouse embryos cultured for 8 days *in* *vitro*. Cultures were double-labeled for the neuronal marker betaIII-tubulin (Tuj1 antibody; red) and Cend1, RanBPM or Dyrk1B (green), respectively, as indicated. The panels on the right show at higher magnification the areas marked by white rectangles in the merged figures. Scale bar: 20 μm. (*b*) Immunoblot analysis of cortical neuron lysates cultured for 8 days *in* *vitro*, confirming the expression of Cend1, RanBPM and Dyrk1B.

As a first step to examine if the protein interactions observed in Neuro 2a cells between Cend1, Dyrk1B and RanBPM may have any relevance in neurons, we analyzed the expression of these molecules in embryonic cortical cultures (Figure **9**). Dissociated cortical neurons were cultured for up to 8 days *in vitro* and were then subjected to double immunofluorescence labeling for the neuronal marker beta III-tubulin and each of the Cend1, RanBPM and Dyrk1B proteins followed by confocal analysis. In accordance with our previous findings in Neuro 2a cells, Cend1 localizes in the cytoplasm of primary neurons while RanBPM partitions primarily in the cytoplasm with residual immunoreactivity also detected in the nucleus. On the other hand, Dyrk1B localization is predominantly nuclear with some immunoreactivity also detected in the cytoplasm (Figure **9a**). These results are compatible with our observations in Neuro 2a cells. The expression of the three molecules in differentiated neurons was also confirmed by immunoblotting (Figure **9b**). 

## Discussion

Regulation of cell cycle exit in neuronal precursors is critical for their timely differentiation and the generation of appropriate numbers of neurons. We have previously shown that Cend1 is a neuronal lineage specific modulator participating in cellular processes connecting cell cycle progression/exit with differentiation of neuronal precursors both *in vitro* and *in vivo* [5,7,10,12,50]. These studies indicated that the negative influence of Cend1 on cell proliferation is mediated via the cyclin D1 pathway that controls the balance between cellular proliferation and differentiation. However, the protein partners interacting directly with Cend1 remained elusive. In this study we identified a direct protein-protein interaction between Cend1 and the scaffolding protein RanBPM and demonstrated that this interaction reverses the anti-proliferative function of Cend1 in mouse neuroblastoma Neuro 2a cells. Further, we asked if Dyrk1B kinase, a previously identified RanBPM partner in lung epithelial cells negatively regulating cyclin D1 [29,36], is also functionally involved in the Cend1 – RanBPM interplay. First we showed that all three proteins are present in brain and in embryonic cultured cortical neurons while RanBPM can form complexes with Cend1 or Dyrk1B. Then we demonstrated that Dyrk1B down-regulates cyclin D1 in Neuro2a cells and that, similarly to Cend1, this effect is reversed upon RanBPM-Dyrk1B co-expression. This occurs because RanBPM facilitates Dyrk1B proteasomal degradation. Finally, we showed that the RanBPM-induced destabilization of Dyrk1B is prevented in the presence of Cend1. Our study thus elucidated a series of functional interactions which resulted in negative or positive regulation of cyclin D1 levels in Neuro 2a cells. Interestingly, these interactions also influenced cell differentiation further supporting a coordinate control of these processes. 

We have previously shown that Cend1 is broadly expressed in the developing neuroepithelium of vertebrates during neurogenesis and that its expression in the chick and mouse CNS is associated with the neuronal, but not the astroglial or oligodendroglial, lineage [8,9]. Indeed, Cend1 is present in precursor cells during the time window they generate neurons, whereas it is downregulated when they shift from a neurogenic to a gliogenic potential [8,9]. Interestingly, Cend1 expression is low in neuronal precursors and higher in differentiated neurons, implying that Cend1 protein levels may influence cell cycle exit and neuronal lineage progression. In agreement, gain-of-function studies in the Neuro 2a cell line or in neural stem/precursor cells cultured *in vitro* as well as in the early chick neural tube *in vivo* provided functional evidence that Cend1 overexpression increases the probability that cells exit the cell cycle and differentiate towards a neuronal pathway [5,6,12]. An opposite phenotype was obtained by Cend1 knock-down in neural precursors or by Cend1 ablation in knock-out mice [5,10]. Recently, Weng et al. (2013) presented new evidence confirming the involvement of Cend1 in neuronal differentiation. These authors identified Cend1 as an Ahi1-interacting partner and showed that loss of Ahi1 reduces the levels of Cend1 in Ahi1 knock-out mice which show defective neuronal differentiation [13]. 

Increase in Cend1 expression is functionally associated with down-regulation of cyclin D1 protein levels in proliferating cells both *in vitro* and *in vivo* [5,7,12]. The cyclin D1 pathway is critical for cell cycle progression/exit in neuronal precursors, also contributing to survival of newborn neurons [51,52]. Here we demonstrated that functional interaction of RanBPM with Cend1 was sufficient to increase cyclin D1 levels in Neuro 2a cells, even above those in control cycling cells, with a concomitant increase in BrdU incorporation indicative of enhanced cell proliferation (Figure 3). The interaction of RanBPM with Cend1 stabilized cyclin D1 in the nucleus to drive cells through the G1-to-S phase of the cell cycle, as shown by BrdU incorporation experiments. Biochemical studies are currently in progress to elucidate the molecular mechanism by which this occurs. We have previously shown that Cend1 over-expression or knock-down studies in Neuro 2a cells but also in the chick neural tube as well as in mice *in vivo*, affect cyclin D1 levels [9,10,12]. The negative influence of Cend1 on cell proliferation has been previously shown to be mediated through the p53/cyclin D1/pRb signalling pathway that controls the balance between cell cycle progression and exit [11,12], while its neuronal differentiation-promoting activity involves downregulation of Notch signalling and activation of the proneural genes network [7,10]. It is possible that the interaction of Cend1 with RanBPM interferes with these pathways.

Of relevance to our present findings, RanBPM has been recently implicated in cell cycle progression of neuronal precursors [28] while it has also been reported to interact with the homeodomain-interacting protein kinase-2 (HIPK2) and the dual specificity tyrosine regulated kinase Dyrk1B both of which affect cell cycle progression/exit [29,53]. As Dyrk1B has been shown to target directly cyclin D1 [36], we chose to incorporate this kinase in our study and investigate a potential mechanism involving Cend1, RanBPM and Dyrk1B in cell cycle progression/exit. Dyrk1B has a strong negative effect on cyclin D1 in Neuro 2a cells resulting in its export from the nucleus to the cytoplasm (Figure 5). This function is dependent on the kinase activity of Dyrk1B as it is repressed by the specific Dyrk kinase inhibitor harmine (Figure 5). This mechanism is reminiscent of the activity of Dyrk1B in proliferating lung epithelial cells where it also causes cell cycle arrest [36]. In these cells Dyrk1B appears to exert its function by directly phosphorylating cyclin D1 on threonine 288, which correlates with its export from the nucleus to the cytoplasm and subsequent degradation [36]. Interestingly, we observed that RanBPM facilitates Dyrk1B nucleocytoplasmic translocation and proteasomal degradation in Neuro 2a cells resulting in stabilization of cyclin D1 protein in the nucleus (Figures 6 and 7). Such a function is in line with previous observations showing that RanBPM plays a regulatory role in protein stability and lifetime as well as in physiological turnover of some of its interactors, as for example the nuclear transcription factor p73 and the mammalian lethal giant larvae-1 (Mgl-1) oncoprotein [54,55]. Earlier studies by Zou and colleagues have indicated that the interaction of RanBPM with Dyrk1B inhibits its kinase activity [29]. As these results were obtained by *in vitro* kinase assays with GST-fusion proteins in the absence of a cellular context, the authors could not have observed a RanBPM-induced proteasomal degradation of Dyrk1B. Our findings and those of Zou et al. (2003) are complementary and suggest a dual effect of RanBPM on Dyrk1B in blocking its kinase activity and facilitating its degradation by the 26S-proteasome. 

The RanBPM-dependent degradation of Dyrk1B could be prevented by Cend1, as shown here, resulting in segregation of Dyrk1B in the nucleus of triple transfected Neuro 2a cells co-expressing RanBPM, Dyrk1B and Cend1 (Figure 8). This led again to a significant reduction in cyclin D1 levels, comparable to those seen in Neuro 2a cells transfected with Dyrk1B alone (Figure 7). These findings suggest that Cend1 and Dyrk1B may compete for binding to RanBPM, although located in different cellular compartments. RanBPM is known to shuttle between the nucleus and the cytoplasm in order to interact with its multiple partners and exert its diverse functions [27,56]. It is possible that Cend1 preferentially binds to RanBPM in the cytoplasm, thus preventing its interaction with Dyrk1B in the nucleus. Notably, the interactions between these three proteins also affect the differentiation of Neuro 2a cells (Figure 9). 

The expression of Cend1, RanBPM and Dyrk1B in brain and their localization in cultured embryonic cortical neurons, indicate a potential biological significance for this tripartite interaction in cell cycle progression/exit and differentiation of neuronal precursors. Recent observations indicate that Dyrk1B protein levels are reduced in Cend1 knock-out mice (Tsioras, Gaitanou and Matsas, unpublished data), which exhibit a delay in cell cycle exit of cerebellar granular neuron precursors, impairment in Purkinje neuron differentiation, ataxic gait and deficits in motor coordination [10]. Interestingly, cerebellar development is disrupted in Joubert syndrome, also characterized by ataxic motor behaviour [57,58]. Therefore association of Cend1 with Ahi1 [13] which is implicated in the pathogenesis of Joubert syndrome and, additionally, has been reported as a susceptibility gene for schizophrenia and autism [18-23], has opened new insights regarding the function of Cend1 and its possible implication in neurodevelopmental disorders. In future studies it will be interesting to examine whether the Cend1/RanBPM/Dyrk1B interaction operates in neuronal cells and/or their precursors and whether such a mechanism is associated with the Ahi1 signaling pathway.

## Supporting Information

Figure S1 ***In house* generated rabbit polyclonal anti-mouse Cend1 antibody tested by immunoblotting.** Polyclonal antiserum against GST-Cend1 chimeric protein (1) before depletion of anti-GST antibodies (2), after depletion of anti-GST antibodies by immunopurification on nitrocellulose strips containing GST protein and (3) after immunopurification on Cend1-containing nitrocellulose strips. (4) Rabbit polyclonal anti- pig Cend1 antibody. All antibodies were tested in dilution 1:1000 in mouse brain homogenate (60 μg/ lane).
(DOCX)

Figure S2 **Fractionation analysis of Neuro 2a cells transiently transfected with Dyrk1B, RanBPM or Cend1 and detection of cyclin D1 by immunoblotting.** Cyclin D1 is detected in the cytoplasmic fraction (C) of Dyrk1B transfected cells while it is preferentially distributed in the cytoplasmic fraction of Cend1-transfected cells. On the other hand, in RanBPM transfected cells, cyclin D1 is mainly detected in the nuclear fraction (N), as in control non-transfected cells (not shown). Fraction purity was checked using anti-GAPDH and anti-PH3 antibodies, respectively.
(DOCX)

Figure S3 **Quantitative real time RT-PCR analysis of Dyrk1B and cyclin D1 mRNA levels.** (*a*, *b*) Neuro 2a cells transiently transfected with Dyrk1B cDNA and allowed for expression 16 and 48 h. CTL, control non-transfected cells. **p <0.01, n= 3. (*c*, *d*) Neuro 2a cells co-transfected with Dyrk1B and increasing amounts of RanBPM, 16h post-transfection (*e*, *f*) Neuro 2a cells co-transfected with Dyrk1B and increasing amounts of GFP, 16h post-transfection.
(DOCX)
