# Supplementary figures and images for: Functional Interactions between BM88/Cend1, Ran-Binding Protein M and Dyrk1B Kinase Affect Cyclin D1 Levels and Cell Cycle Progression/Exit in Mouse Neuroblastoma Cells
Source: PLoS One. 2013 Nov 28;8(11):e82172. doi: 10.1371/journal.pone.0082172 (PMC3842983; doi:10.1371/journal.pone.0082172)

SUPPLEMENTAL DATA

**Figure S1**


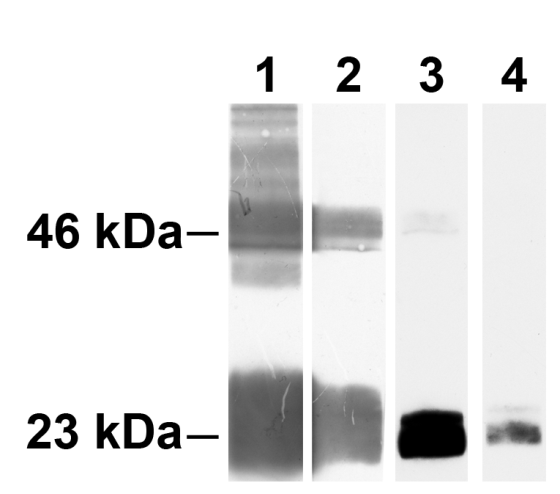

Supplement: Figure S1 — In house generated rabbit polyclonal anti-mouse Cend1 antibody tested by immunoblotting. Polyclonal antiserum against GST-Cend1 chimeric protein (1) before depletion of anti-GST antibodies (2), after depletion of anti-GST antibodies by immunopurification on nitrocellulose strips containing GST protein and (3) after immunopurification on Cend1-containing nitrocellulose strips. (4) Rabbit polyclonal anti- pig Cend1 antibody. All antibodies were tested in dilution 1:1000 in mouse brain homogenate (60 μg/ lane). (DOCX) [file pone.0082172.s001.docx]

SUPPLEMENTAL DATA

**Figure S2**


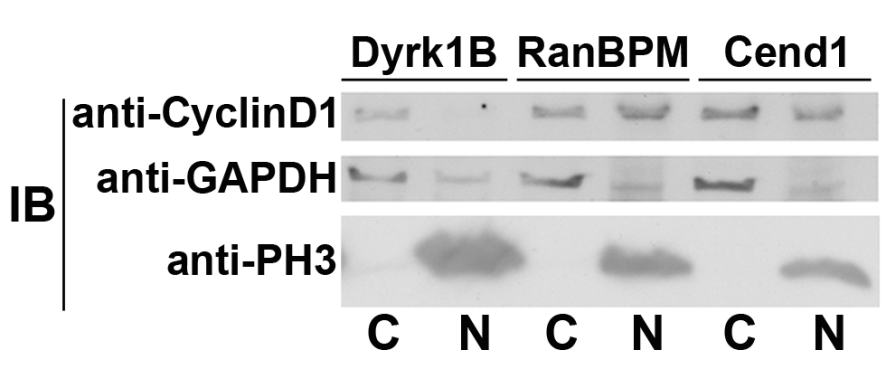

Supplement: Figure S2 — Fractionation analysis of Neuro 2a cells transiently transfected with Dyrk1B, RanBPM or Cend1 and detection of cyclin D1 by immunoblotting. Cyclin D1 is detected in the cytoplasmic fraction (C) of Dyrk1B transfected cells while it is preferentially distributed in the cytoplasmic fraction of Cend1-transfected cells. On the other hand, in RanBPM transfected cells, cyclin D1 is mainly detected in the nuclear fraction (N), as in control non-transfected cells (not shown). Fraction purity was checked using anti-GAPDH and anti-PH3 antibodies, respectively. (DOCX) [file pone.0082172.s002.docx]

SUPPLEMENTAL DATA

**Figure S3**


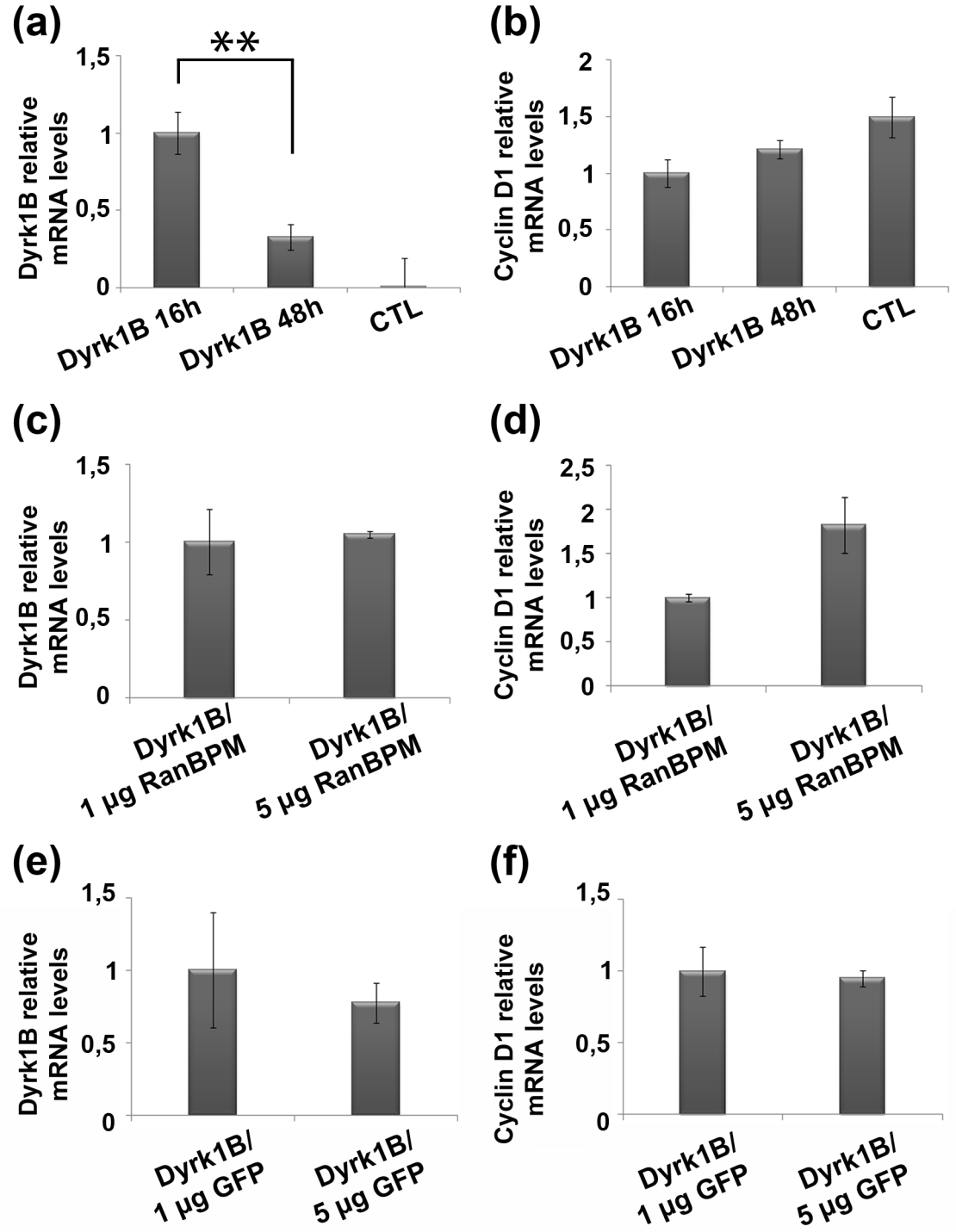

Supplement: Figure S3 — Quantitative real time RT-PCR analysis of Dyrk1B and cyclin D1 mRNA levels. (a, b) Neuro 2a cells transiently transfected with Dyrk1B cDNA and allowed for expression 16 and 48 h. CTL, control non-transfected cells. **p <0.01, n= 3. (c, d) Neuro 2a cells co-transfected with Dyrk1B and increasing amounts of RanBPM, 16h post-transfection (e, f) Neuro 2a cells co-transfected with Dyrk1B and increasing amounts of GFP, 16h post-transfection. (DOCX) [file pone.0082172.s003.docx]
